# Comparing Online and On-Site Cognitive Behavior Therapy in Major Depressive Disorder: Protocol for a Noninferiority Randomized Controlled Trial

**DOI:** 10.2196/29726

**Published:** 2022-04-08

**Authors:** Paul Ritvo, David Gratzer, Yuliya Knyahnytska, Abigail Ortiz, Clarice Walters, Joel Katz, Judith Laposa, Christopher Baldissera, Noah Wayne, Donna Pfefer-Litman, George Tomlinson, Zafiris Daskalakis

**Affiliations:** 1 School of Kinesiology and Health Science York University Toronto, ON Canada; 2 Centre for Addiction and Mental Health Toronto, ON Canada; 3 Department of Medicine, University Health Network and Mt. Sinai Hospital Institute of Health Policy, Management and Evaluation University of Toronto Toronto, ON Canada; 4 NexJ Health, Inc. Toronto, ON Canada; 5 Department of Psychiatry University of California, San Diego San Diego, CA United States

**Keywords:** online intervention, randomized controlled trial, major depressive disorder

## Abstract

**Background:**

The incidence of mental health disorders in Canada is increasing with costs of CAD $51 billion (US $40 billion) per year. Depression is the most prevalent cause of disability while cognitive behavioral therapy (CBT) is the best validated behavioral depression treatment. CBT, when combined with mindfulness meditation (CBT-M), has strong evidence for increased efficacy. While randomized controlled trials (RCTs) have demonstrated online CBT-M efficacy, comparisons with in-office delivery are lacking.

**Objective:**

The aim of this research is to assess whether online group CBT-M (with standard psychiatric care) is non-inferior in efficacy and more cost-effective than office-based, on-site group CBT-M at post-intervention and 6-months follow-up in major depressive disorder. The study will also assess whether digitally recorded data (ie, online workbooks completed, Fitbit step count, and online text messages) predict depression symptom reduction in online participants.

**Methods:**

This single-center, two-arm, noninferiority RCT employs assessor-blinded and self-report outcomes and economic evaluation. The research site is the Centre for Addiction and Mental Health (Toronto), a research-based psychiatry institution where participants will be identified from service wait lists and through contacts with other Toronto clinics. Inclusion criteria are as follows: (1) aged 18-60 years, any ethnicity; (2) Beck Depression Inventory-II (BDI-II) of mild severity (score ≥14) with no upper severity limit; (3) Mini-International Neuropsychiatric Interview-confirmed, psychiatric major depressive disorder diagnosis; (4) fluent in English. All patients are diagnosed by staff psychiatrists. Exclusion criteria are as follows: (1) receipt of weekly structured psychotherapy; (2) observation of Diagnostic and Statistical Manual of Mental Disorders (5th Edition) criteria for severe alcohol or substance use disorder (in past 3 months), borderline personality disorder, schizophrenia (or other primary psychotic disorder), bipolar disorder, or obsessive-compulsive disorder; (3) clinically significant suicidal ideation (imminent intent or attempted suicide in the past 6 months); and (4) treatment-resistant depression. All participants receive standard psychiatric care, experimental participants receive online group CBT-M, and controls receive standard care in-office group CBT-M. The online group program (in collaboration with NexJ Health, Inc) combines smartphone and computer-accessed workbooks with mental health phone counselling (16 hours in 16 weeks) that coordinates software interactions (eg, secure text messaging and Fitbit-tracked walking). The primary outcome is BDI-II, and secondary outcomes are anxiety (Beck Anxiety Inventory), depression (ie, Quick Inventory of Depressive Symptomatology and 17-item Hamilton Depression Rating Scale), mindfulness (Five-Facet Mindfulness Questionnaire), quality of life (European Quality of Life Five Dimension), and pain (Brief Pain Inventory).

**Results:**

Based on prior studies with the BDI-II and 80% power to reject an inferiority hypothesis with a 1-sided type I error rate of 5%, a sample of 78 per group is adequate to detect small-to-medium–effect sizes.

**Conclusions:**

This study assesses online CBT-M efficacy and noninferiority in relation to in-person CBT, and the cost-effectiveness of both interventions.

**Trial Registration:**

ClinicalTrials.gov NCT04825535; https://www.clinicaltrials.gov/ct2/show/NCT04825535

**International Registered Report Identifier (IRRID):**

DERR1-10.2196/29726

## Introduction

Mental health disorders are Canada’s most costly chronic health problem for both health systems and patients, and they are increasing in incidence. The current economic costs of mental health are estimated at CAD $51 billion annually (US $40 billion), with CAD $42.3 billion (US $33 billion) in direct costs to the Canadian health care system [1]. Depression is a commonly diagnosed mental health disorder, representing the most prevalent cause of disability worldwide [2]. Cognitive behavioral therapy (CBT) is the best validated psychotherapy for the treatment of depression, with decades of research demonstrating its efficacy [3]. Despite demonstrated efficacy, many individuals are not able to access adequate CBT treatment due to the limitations of face-to-face delivery. Improving access to CBT services is crucial to overcoming the treatment barriers (including geographic distance, cost, time, and perceived stigma) that currently prevent access to necessary mental health care for individuals with depression [4-6]. Recent research projects have focused on internet-based CBT (with internet-delivered or telephone-based interventions) as a means of delivering cost-effective treatment to populations that are unable to access high-quality psychotherapy services within traditional in-office environments. The structured and goal-directed modules of CBT are well suited to online delivery, and numerous randomized controlled trials (RCTs) have indicated that internet-delivered CBT is highly effective in symptom reduction and depression remission [7-9]. In recent years, CBT has been integrated with mindfulness meditation (CBT-M) following strong evidence for the increased efficacy when CBT and mindfulness meditation are combined [10]. While other trials support online CBT and mindfulness efficacy versus control groups [11-18], no prior trial, to our knowledge, has compared the efficacy and cost-effectiveness of online group CBT-M versus standard office-based group CBT-M.

Previous noninferiority RCTs have indicated that guided online CBT can be at least as effective as in-person CBT for the treatment of depression; however, these studies are limited by the use of small, nonclinical samples and do not include cost-effectiveness comparisons [19-21]. Research is now required with an adequate sample size and in-depth cost comparisons to fully establish noninferiority and cost-effectiveness [22]. This study has been registered with ClinicalTrial.gov (NCT04825535).

## Methods

### Aim

This study has the 3 following aims: (1) to evaluate whether online group CBT-M (with standard psychiatric care) is noninferior in efficacy compared with office-based group CBT-M (with standard care) as measured by the BDI-II (Beck Depression Inventory-II) score in the treatment of adults with major depressive disorder (MDD) [23]; (2) to evaluate whether online group CBT-M is more cost-effective than office-based group CBT-M post intervention and at 6 months post-intervention follow-up; (3) to assess, within the online group CBT-M intervention group, whether digitally recorded adherence data (ie, online workbooks completed, Fitbit tracked steps, online text messages exchanged, and phone sessions completed) predict outcome benefits as indicated by the changed BDI-II score [23].

### Ethical Considerations, Recruitment, and Randomization

The study has been approved by the Research and Ethics Board at the Centre for Addiction and Mental Health (CAMH) (reference 087/2020). The participants will be identified from the wait lists within the CAMH General Adult Psychiatry and Health Systems Division, which annually services thousands of MDD patients. The attending physician or clinician identifies prospective participants and asks for their verbal consent to receive more information about the research. Once the patient’s attending physician or clinician obtains verbal consent for more information about the research project, he or she can refer eligible participants to the research analyst who explores interest with the participant, and after an expressed interest, reviews and explains the study. Eligibility screening and written consent are undertaken in person prior to randomization. The study biostatistician performs electronic randomization with study IDs blindly assigned to intervention (online CBT-M plus standard psychiatric care) and control (office-based group CBT-M plus standard psychiatric care) groups. The study ID information with the respective group allocation will be transferred onto cards placed in opaque, individual, sealed envelopes. After a participant completes baseline questionnaires, the research analyst opens the next envelope in sequence to determine group allocation and study ID. Based on the results of previously successful RCTs, including the one conducted at the identical study site, we will recruit 100 participants per group or 200 overall (N=200). One-sided type 1 error rate is set at 5%. We chose a margin of 3 and a correlation of 0.70, indicating a sample size of 78 per group in 2 groups, which is more than adequate for the detection of small-to-medium effect sizes. The 16-week intervention entails 16 weekly group CBT in-person sessions (control group) (1.5 hours in duration) and 16 weekly, phone-based navigator-health coach sessions (16 hours in duration per subject in group or individual formats). Participants are assigned to review 1-2 workbooks and related videos per week (16-32 workbooks per 16 weeks). Assessments take place at post-intervention and 6 months follow-up. Given the recruitment, consent, and intervention target of 48 participants per year, we expect to complete the study within 2.5 to 3 years.

#### Inclusion Criteria

The inclusion criteria are as follows: (1) individuals 18-60 years of age; (2) BDI-II of at least mild severity (score ≥14) with no upper severity limit [23]; (3) MINI (Mini-International Neuropsychiatric Interview)-confirmed diagnosis of MDD [24]; and (4) fluency in English. All participants are diagnosed by a CAMH staff psychiatrist with diagnosis confirmed via the MINI [24], administered at the screening visit.

#### Exclusion Criteria

The exclusion criteria are as follows: (1) individuals currently receiving weekly structured psychotherapy; (2) individuals who meet DSM-V (Diagnostic and Statistical Manual of Mental Disorders, 5th Edition) criteria for severe alcohol or substance use disorder (in the past 3 months), borderline personality disorder, schizophrenia, or any other primary psychotic disorder, bipolar disorder, or obsessive-compulsive disorder; (3) individuals who manifest clinically significant suicidal ideation defined as imminent intent or attempted suicide (in the past 6 months); and (4) individuals who are judged to have treatment-resistant depression, as defined by failure in at least 2 trials of antidepressant medications or a course of psychotherapy during the current depressive episode [25].

### Interventions

#### Online Intervention

The online group CBT-M program combines online-accessed workbooks with phone-based navigator coaching that assists with the coordination and support of multiple software interactions (eg, secure text messaging, Fitbit tracked walking, and food monitoring via photography). Each participant is loaned a Fitbit HR Charge 4 (Fitbit Inc), which assesses physical steps and 24-hour heart rate, averaged in 5-second intervals. Intervention content builds on 6 online RCTs with students [26-32], 1 RCT with adults with type 2 diabetes [11,33,34] (where significant mental health and blood glucose benefits resulted), and 1 online RCT with youth diagnosed with MDD [22]. The content contains 24 workbook chapters on multiple topics derived from the focus group study (eg, Living By Your Truths, Overcoming Wired-ness and Tired-ness, Mindfulness and Relationships, Loss and Grief, Resilience, Befriending Ourselves, Befriending Your Body with Exercise, Body Image, Intimacy, Forgiveness, Overcoming Procrastination, Dealing with Negative Moods, Stress Resilience, Reducing Performance Anxiety, and Cultivating Inspiration), addressed in sequences mutually agreed on by the participants and navigator-coaches.

The online CBT-M groups address structured tasks that elevate mood and decrease anxiety. Depressive social withdrawals are identified and reflected on while corrective emotional experiences are elicited, structured, and supported. Healthy internet interactions are reinforced by the web-based program content (24 workbooks and 56 videos), with priorities guided by participant interests. While internet-based contacts are sometimes distracting “escapes” (eg, Netflix movies and video game playing), they can be productive, socially engaging events (eg, TED Talks, CBT discussion, and mindfulness instructions) where role-modeling coordinates with learning. Group members stimulate and reinforce health behavior adoptions (exercise, CBT, mindfulness, sleep hygiene, diet, and text message encouragement) and transformations from distractive media fixations to positive learning and enjoyment. Internet-based contacts integrate with face-to-face professional and familial contacts and social intimacy development. Group online CBT-M tasks emphasize applying CBT-M methods in accord with content themes, which are decatastrophizing, overcoming perfectionism, sound sleep, self-befriending, courage development, forgiveness, interpersonal mindfulness, and autonomous generosity. This progression coordinates physical and cognitive changes that integrate interpersonal contacts with self-management. The tracking of walking exercise and heart rate (HR), on a 24-hour basis, captures HR elevations that reflect episodes of anxiety and negative affect (elevated HR with minimal movement). There is selective sharing of Fitbit bio-behavioral data with designated staff and group members. Reduced HR often accompanies mindfulness practice while mildly elevated HR is closely associated with walking (ie, movement-affected HR). Self-modification goals involve improving autonomic nervous system balance through appropriate exercise, improved sleep hygiene, and restorative sleep [35].

#### Office-Based Intervention

The on-site, usual-care CBT group follows the structure of the “Mind Over Mood” workbook in reviewing CBT concepts and procedures [36]. A series of worksheets assist participants in differentiating moods from thoughts and situational influences, leading to modifications of thinking, behavior, emotion, and mood.

Difficulties with cognitive change are assumed to be associated with self-acceptance deficits and negative mood dominance. Increased self-acceptance is linked with mindfulness experiences, which involve “more focusing on the present moment” and on nonjudgmental acceptance of ongoing experience. Mindful breathing awareness is described in content on “balanced deep breathing” and in tension releases achieved via progressive muscle relaxation practice [37].

An emphasized structure is the automatic thought record by which negative, disturbing thoughts are identified, and attention directed to alternate thoughts evaluated as clearer and less distorted. These transitions are more difficult when immersions in negative moods diminish the confidence in finding a “better” thought. Accordingly, self-efficacy is an important goal, emphasized in exchanges of support between group members and in enhanced experiences of self-acceptance.

Behavioral activations are structured for the development of increased awareness and observations of how self-control resources elicit gratifying activities. On the basis of behavioral activation practices, cognitive restructuring strategies utilize the automatic thought record or experiential approximations of the automatic thought record [38].

Another major component of group CBT-M at CAMH involves examinations of core beliefs where multiple techniques are employed (eg, self-observation logs and historical self-reviews) in the group setting and in individual homework assignments between sessions [38].

### Hypothesis

Online group CBT-M will be noninferior to standard, office-based group CBT in the treatment of MDD (as indicated by BDI-II score change) when online and office-based treatment groups are compared, using both intention-to-treat and per-protocol analyses. Online group CBT-M will be more cost-effective than standard, office-based group CBT, as measured by cumulative costs and quality-adjusted life years (QALY) calculated in the cost utility analysis. Within the online CBT-M intervention participant group, digitally recorded adherence data will predict outcome benefits (as reflected in the BDI-II score change). Adherence over time will be compared within each participant using a generalized estimating equation logistic regression model with an autoregressive 1 (AR1) correlation structure.

### Outcome Measures

#### Primary Outcome

The primary outcome measure is the BDI-II [23]. It is a 21-item self-report scale, measuring symptoms of depression with a 4-point Likert scale (0 to 3), with higher scores indicating more severe depressive symptoms. The customary cut-off scores for mild depression are 14 to 25, moderate depression 26 to 30, and severe depression 31 and greater.

#### Secondary Outcomes

The secondary outcomes assess the following: anxiety (Beck Anxiety Inventory) [39]; depression (QIDS [Quick Inventory of Depressive Symptomatology] [40] and HDRS-17 [17-item Hamilton Depression Rating Scale], consisting of an interview with a blinded-assessor rater) [41]; mindfulness (FFMQ [Five-Facet Mindfulness Questionnaire]) [42]; quality of life (EQ-5D [European Quality of Life Five Dimension]) [43,44]; patient costs (health care cost diary for major depression) [45]; and pain (brief pain inventory) [46].

All self-report measures and the HDRS-17 interview are carried out at the same CAMH mood and anxiety research clinic in identical assessment rooms. The HDRS-17 interviewer-assessor is blinded to intervention and control conditions for the trial duration and is experienced in the blinded-assessor role with similar conditions conducted at the identical study site used in this trial [22].

## Results

### Analyses

#### Primary Outcome

The primary analysis will be a Bayesian analysis of covariance (ANCOVA), with change in BDI-II between baseline and 16 weeks as the outcome and 2 covariates, the baseline score and the intervention group variable, coded as “O” (for online) and “I” (for in-office). We refer to the parameter of interest as ∆, the baseline-adjusted difference between the 2 groups in the change in BDI-II from baseline to 16 weeks, coded such that ∆<0 means that the online group has a smaller decrease in the level of depression over the course of the study. Then, if ∆>-3, the improvement in the online group is at most 3 points worse than the improvement in the in-office group, and the online group is noninferior to the in-office group. Using noninformative priors for all study parameters in the ANCOVA model, we will compute and plot the full posterior distribution of ∆, presenting the lower 95% credible interval for ∆, and then calculating the posterior probability that ∆>-3; in other words, the probability (after observing the study data) that online CBT is noninferior, according to our definition. One advantage of a Bayesian approach is that it allows the assigning of posterior probabilities of noninferiority at other margins; notably, other margins near 3 may have equal levels of evidentiary support. For example, without a penalty for multiple testing, we can compute the probability that ∆>-2 or ∆>-4. Another advantage of the Bayesian approach is that it involves a more useful presentation of results than a simple confidence interval or *P* value. Finally, the outputs of the Bayesian model can be used as probabilistic inputs for the economic analysis. Since this is a noninferiority approach, the primary analysis will be per protocol, which, in the presence of intervention nonadherence, is more conservative than intention-to-treat (ie, less likely to conclude that groups are similar when they are not). Three secondary analyses of the primary outcome will be conducted. First, we will make the above between-group comparison of the changes in BDI-II scores from baseline to each follow-up time point. Secondly, we will perform an intention-to-treat analysis, including outcomes on participants who were nonadherent to their program (standard care CBT vs CBT online) using established cut-offs. Finally, to assess for sensitivity to random imbalance in baseline characteristic, the following covariates will be added to the ANCOVA model: age, baseline anxiety, baseline depression (HDRS-17), baseline pain, and baseline mindfulness. If more than 5% of the participants included in the per protocol are missing outcome data, that analysis will use multiple imputations within the Bayesian model with full baseline covariate data used to impute missing outcomes. In a second approach, we will assume that missing follow-up data are missing not at random and replace missing values by values randomly sampled from the upper half (high BDI-II) of their predictive distribution. This represents the assumption that those who have no follow-up BDI-II have values lower than the median predicted by their baseline characteristics. This will be repeated within the intention-to-treat analysis, where missing outcome data will be more common.

#### Secondary Outcomes

A similar modeling approach will be used for each secondary outcome (Beck Anxiety Inventory, QIDS, and HDRS-24), mindfulness (FFMQ [Five-Facet Mindfulness Questionnaire]), pain (brief pain inventory), health care cost diary for major depression, and quality of life (EQ-5D). While noninferiority margins per outcome are nonexistent, we will present probabilities for a range of margins based on fractions of the minimally clinical important difference for these scales (where available) or fractions of a standard deviation. All primary and secondary outcomes will be analyzed as continuous variables, with outcomes modelled as normal, *t*, rescaled beta, log-normal, or gamma distributions, whichever is most appropriate.

#### Postintervention Period Outcomes

We will examine BDI-II changes over time across the 2 intervention delivery models during a 6-month post-intervention period. To give a quantitative overview of changes between and within groups over 6 months, all study outcomes will be assessed using linear mixed effects models incorporating within-person correlation. Mixed effects models allow for including partial data (eg, from participants with missed visits or who dropped out prematurely) while accounting for repeated within-person data [47]. The baseline and follow-up scores (up to 10 months from baseline) will form the dependent variable and intervention group, time, and the time-treatment interactions will be the independent variables. Within-person correlation of residuals will be handled by an AR1 error structure. Through the examination of estimated model parameters and their 95% confidence intervals, we can assess whether changes over time are similar between groups and whether differences between groups at any given time are clinically important.

### Adherence

Multiple analyses are used to assess adherence in the online group CBT-M program and the relationship of adherence to both time and potential predictors. Adherence is cumulatively and quantitatively defined as a binary variable (adherent or nonadherent). We will assess adherence over 2 distinct periods—0 to 2 months and 2 to 4 months—so each participant has up to 2 adherence measures. Our first analysis of adherence will simply be the calculation of percentages adhering in each of the time windows. The chi-squared or Fisher exact tests will compare adherence within each of the time windows. All further analyses will be carried out on the sequence of 2 adherence measures on each participant using a generalized estimating equation logistic regression model with an AR1 correlation structure. A series of models will be run, which is as follows: to check adherence changes over time, we will use time as a categorical predictor variable and assess its importance using a Wald test; to examine predictors of adherence, variables (steps per day or HR, workbooks completed, text messages exchanged, videos watched, and phone counselling calls completed) measured at the start of each of the 2 time periods will be added as predictors for the corresponding adherence outcome. These time-varying predictors help us assess whether the most recent assessment of a given predictor predicts adherence over the following 2 months.

### Missing Data

Missing data may occur when participants miss an assessment or outcome measure but continue the study participation or because participants drop out prematurely. In the first case, regression-based imputation at the individual patient level will be used to impute the missing outcomes. In the second case, imputing data using bottom quintile scores of responders will be used as a worst-case sensitivity analysis. Loss to follow-up is unavoidable in MDD studies and can reflect poor intervention response. It was a relevant comparison variable in the recently completed RCT where loss to follow-up in the intervention arm was 10%, compared to 60% in the standard psychiatry control arm [22]. While differences may be more modest in the proposed trial (eg, 40% in group in-office CBT versus 10% in group online CBT), they will be carefully monitored and, as previously mentioned, represented in the cost-effectiveness and intention-to-treat analyses.

### Cost-effectiveness

We will conduct a full economic evaluation following the design of a cost-utility analysis conducted from a societal and health care consumer or payer’s perspective. We will adopt the following time horizons: within trial (4 and 10 months) and the lifetime of the trial cohort. A within-trial cost utility analysis will focus only on interventions directly evaluated in the trial. We will estimate costs for (1) the interventions; (2) physician services; (3) emergency department visits and hospitalizations; (4) outpatient diagnostic tests; (5) drugs, including those unrelated to MDD; (6) home care; (7) long-term care; (8) out-of-pocket costs; and (9) productivity costs. The intervention cost will be estimated by estimating the value of time of those administering the intervention, facility-use costs, and device or equipment costs, amortized over an appropriate period. Resource utilization, out-of-pocket costs, and productivity costs will be estimated using a patient cost diary. We will assess health outcome data in QALY using the EQ-5D at each time point [48-52]. Cumulative costs and QALY will be estimated and compared in order to calculate the incremental cost utility ratio, and incremental net health benefit. We will plot the cost-effectiveness acceptability curves and confidence ellipses to demonstrate variability in a trial sample and illustrate probability of online CBT-M being cost-effective compared with a standard CBT program at a range of willingness-to-pay thresholds.

## Discussion

If the hypothesized results are obtained, online group CBT-M for the treatment of MDD can be used by health care providers to deliver high-quality psychotherapy to more people in need. Online group CBT-M may be a primary treatment option for individuals currently unable to access standard office-based care due to distance, time, or cost. The cost-effectiveness analysis of online versus office-based group CBT-M will provide policy makers with definitive evidence to appropriately budget for internet-delivered CBT services. Acknowledged study limitations include the lack of blinding of self-report measures other than the interview-delivered HDRS-17 assessment [41].

## Appendix

Multimedia Appendix 1 CONSORT eHEALTH Checklist (V 1.6.1.).

## Abbreviations

ANCOVA: analysis of covariance
AR1: autoregressive 1
BDI-II: Beck Depression Inventory-II
CAMH: Centre for Addiction and Mental Health
CBT: cognitive behavioral therapy
CBT-M: cognitive behavioral therapy with mindfulness
DSM-V: Diagnostic and Statistical Manual of Mental Disorders, 5th Edition
EQ-5D: European Quality of Life Five Dimension
FFMQ: Five-Facet Mindfulness Questionnaire
HDRS: Hamilton Depression Rating Scale
HR: heart rate
MDD: major depressive disorder
MINI: Mini-International Neuropsychiatric Interview
QALY: quality-adjusted life years
QIDS: Quick Inventory of Depressive Symptomatology
RCT: randomized controlled trial
