# Correction: Comparing Online and On-Site Cognitive Behavior Therapy in Major Depressive Disorder: Protocol for a Noninferiority Randomized Controlled Trial

**DOI:** 10.2196/38720

**Published:** 2022-04-21

**Authors:** Paul Ritvo, David Gratzer, Yuliya Knyahnytska, Abigail Ortiz, Clarice Walters, Joel Katz, Judith Laposa, Christopher Baldissera, Noah Wayne, Donna Pfefer-Litman, George Tomlinson, Zafiris Daskalakis

**Affiliations:** 1 School of Kinesiology and Health Science York University Toronto, ON Canada; 2 Centre for Addiction and Mental Health Toronto, ON Canada; 3 Department of Medicine, University Health Network and Mt. Sinai Hospital Institute of Health Policy, Management and Evaluation University of Toronto Toronto, ON Canada; 4 NexJ Health, Inc Toronto, ON Canada; 5 Department of Psychiatry University of California, San Diego San Diego, CA United States

In “Comparing Online and On-Site Cognitive Behavior Therapy in Major Depressive Disorder: Protocol for a Noninferiority Randomized Controlled Trial” (JMIR Res Protoc 2022;11(4):e29726), the following change was made:

In the originally published article, the Conflicts of Interest section inadvertently appeared as follows:

None declared.

In the corrected version, the Conflicts of Interest section has been corrected as follows:

NW is an employee of NexJ Health and holds stock in the company. NexJ Health provides in-kind subscriptions for the digital health platform of NexJ Connected Wellness, which enables the delivery of the CBT-M program and provides health coaching to the participants in the CBT-M intervention group. PR receives in-kind software support from NexJ Health for this investigator-initiated study, funded by the Canadian Institutes of Health Research (CIHR). He also receives research support from NexJ Health through the Digital Health Research Fund administered by the Faculty of Health at York University. ZD has received research and equipment in-kind support for an investigator-initiated study through Brainsway Inc and Magventure Inc. He is also on the scientific advisory board for Brainsway Inc. His work has been supported by the National Institutes of Mental Health (NIMH), Canadian Institutes of Health Research (CIHR), Brain Canada, and Temerty Family Foundation, and Grant Family Foundation.

The correction will appear in the online version of the paper on the JMIR Publications website on April 21, 2022, together with the publication of this correction notice. Because this was made after submission to PubMed, PubMed Central, and other full-text repositories, the corrected article has also been resubmitted to those repositories.

