# Supplementary material for: Comparing Online and On-Site Cognitive Behavior Therapy in Major Depressive Disorder: Protocol for a Noninferiority Randomized Controlled Trial
Source: JMIR Res Protoc. 2022 Apr 8;11(4):e29726. doi: 10.2196/29726 (PMC9022777; doi:10.2196/29726)
Supplement: Multimedia Appendix 1 [file resprot_v11i4e29726_app1.pdf]

CONSORT-EHEALTH (V 1.6.1) - Submission/Publication Form

The CONSORT-EHEALTH checklist is intended for authors of randomized trials evaluating web-based and Internet-based applications/interventions, including mobile interventions, electronic games (incl multiplayer games), social media, certain telehealth applications, and other interactive and/or networked electronic applications. Some of the items (e.g. all subitems under item 5 - description of the intervention) may also be applicable for other study designs.

The goal of the CONSORT EHEALTH checklist and guideline is to be
a) a guide for reporting for authors of RCTs,
b) to form a basis for appraisal of an ehealth trial (in terms of validity)

CONSORT-EHEALTH items/subitems are MANDATORY reporting items for studies published in the Journal of Medical Internet Research and other journals / scientific societies endorsing the checklist.

Items numbered 1., 2., 3., 4a., 4b etc are original CONSORT or CONSORT-NPT (non-pharmacologic treatment) items.
Items with Roman numerals (i., ii, iii, iv etc.) are CONSORT-EHEALTH extensions/clarifications.

As the CONSORT-EHEALTH checklist is still considered in a formative stage, we would ask that you also RATE ON A SCALE OF 1-5 how important/useful you feel each item is FOR THE PURPOSE OF THE CHECKLIST and reporting guideline (optional).

Mandatory reporting items are marked with a red *.
In the textboxes, either copy & paste the relevant sections from your manuscript into this form - please include any quotes from your manuscript in QUOTATION MARKS,
or answer directly by providing additional information not in the manuscript, or elaborating on why the item was not relevant for this study.

YOUR ANSWERS WILL BE PUBLISHED AS A SUPPLEMENTARY FILE TO YOUR PUBLICATION IN JMIR AND ARE CONSIDERED PART OF YOUR PUBLICATION (IF ACCEPTED).
Please fill in these questions diligently. Information will not be copyedited, so please use proper spelling and grammar, use correct capitalization, and avoid abbreviations.

DO NOT FORGET TO SAVE AS PDF _AND_ CLICK THE SUBMIT BUTTON SO YOUR ANSWERS ARE IN OUR DATABASE !!!

Citation Suggestion (if you append the pdf as Appendix we suggest to cite this paper in the caption):
Eysenbach G, CONSORT-EHEALTH Group
CONSORT-EHEALTH: Improving and Standardizing Evaluation Reports of Web-based and Mobile Health Interventions

J Med Internet Res 2011;13(4):e126
URL: http://www.jmir.org/2011/4/e126/
doi: 10.2196/jmir.1923
PMID: 22209829

claricemwalters@gmail.com (not shared) Switch account

Draft not saved

* Required

Your name *

First Last

Paul Ritvo

Primary Affiliation (short), City, Country *

University of Toronto, Toronto, Canada

York University, Toronto, Canada

Your e-mail address *

abc@gmail.com

paul.ritvo@gmail.com

Title of your manuscript *

Provide the (draft) title of your manuscript.

Comparing Online and On-Site Cognitive Behavioural Therapy in Major Depressive Disorder: Non-Inferiority Randomized Controlled Trial

Name of your App/Software/Intervention *

If there is a short and a long/alternate name, write the short name first and add the long name in brackets.

Cognitive Behavioural Therapy (CBT; interventi

Evaluated Version (if any)

e.g. "V1", "Release 2017-03-01", "Version 2.0.27913"

Version 3.9.17

Language(s) *

What language is the intervention/app in? If multiple languages are available, separate by comma (e.g. "English, French")

English, French

URL of your Intervention Website or App

e.g. a direct link to the mobile app on app in appstore (itunes, Google Play), or URL of the website. If the intervention is a DVD or hardware, you can also link to an Amazon page.

https://apps.apple.com/us/app/nexj-health-coach/id1047247250

URL of an image/screenshot (optional)

Your answer

Accessibility *

Can an enduser access the intervention presently?

- ( ) access is free and open
- (•) access only for special usergroups, not open
- ( ) access is open to everyone, but requires payment/subscription/in-app purchases
- ( ) app/intervention no longer accessible
- ( ) Other:

Primary Medical Indication/Disease/Condition *

e.g. "Stress", "Diabetes", or define the target group in brackets after the condition, e.g. "Autism (Parents of children with)", "Alzheimers (Informal Caregivers of)"

Major Depressive Disorder (in adults ages 18-6

Primary Outcomes measured in trial *

comma-separated list of primary outcomes reported in the trial

Change in depressive symptoms as measured

Secondary/other outcomes

Are there any other outcomes the intervention is expected to affect?

The secondary outcome measures are anxiety (Beck Anxiety Inventory (BAI)), depression (Quick Inventory of Depressive Symptomatology; (QIDS) and the 24-item Hamilton Depression Rating Scale (HDRS-24); in addition to the the BDI-II), mindfulness (Five-Facet Mindfulness Questionnaire (FFMQ)), quality of life (Euro Qol-5D), patient costs (Health Care Cost Diary for Major Depression), and pain (Brief Pain Inventory (BPI)).

Recommended "Dose" *

What do the instructions for users say on how often the app should be used?

- (●) Approximately Daily
- ( ) Approximately Weekly
- ( ) Approximately Monthly
- ( ) Approximately Yearly
- ( ) "as needed"
- ( ) Other:

Approx. Percentage of Users (starters) still using the app as recommended after 3 months *

- (●) unknown / not evaluated
- ( ) 0-10%
- ( ) 11-20%
- ( ) 21-30%
- ( ) 31-40%
- ( ) 41-50%
- ( ) 51-60%
- ( ) 61-70%
- ( ) 71%-80%
- ( ) 81-90%
- ( ) 91-100%
- ( ) Other:

Overall, was the app/intervention effective? *

- ○ yes: all primary outcomes were significantly better in intervention group vs control
- ○ partly: SOME primary outcomes were significantly better in intervention group vs control
- ○ no statistically significant difference between control and intervention
- ○ potentially harmful: control was significantly better than intervention in one or more outcomes
- ○ inconclusive: more research is needed
- ◉ Other: This is a protocol paper and as such participant data from the interve

Article Preparation Status/Stage *

At which stage in your article preparation are you currently (at the time you fill in this form)

- ○ not submitted yet - in early draft status
- ○ not submitted yet - in late draft status, just before submission
- ○ submitted to a journal but not reviewed yet
- ○ submitted to a journal and after receiving initial reviewer comments
- ◉ submitted to a journal and accepted, but not published yet
- ○ published
- ○ Other:

Journal *

If you already know where you will submit this paper (or if it is already submitted), please provide the journal name (if it is not JMIR, provide the journal name under "other")

- ○ not submitted yet / unclear where I will submit this
- ◉ Journal of Medical Internet Research (JMIR)
- ○ JMIR mHealth and UHealth
- ○ JMIR Serious Games
- ○ JMIR Mental Health
- ○ JMIR Public Health
- ○ JMIR Formative Research
- ○ Other JMIR sister journal
- ○ Other:

Is this a full powered effectiveness trial or a pilot/feasibility trial? *

- ○ Pilot/feasibility
- ◉ Fully powered

Manuscript tracking number *

If this is a JMIR submission, please provide the manuscript tracking number under "other" (The ms tracking number can be found in the submission acknowledgement email, or when you login as author in JMIR. If the paper is already published in JMIR, then the ms tracking number is the four-digit number at the end of the DOI, to be found at the bottom of each published article in JMIR)

- ○ no ms number (yet) / not (yet) submitted to / published in JMIR
- ◉ Other: #29726

TITLE AND ABSTRACT

1a) TITLE: Identification as a randomized trial in the title

1a) Does your paper address CONSORT item 1a? *

I.e does the title contain the phrase "Randomized Controlled Trial"? (if not, explain the reason under "other")

- (●) yes
- ( ) Other:

1a-i) Identify the mode of delivery in the title

Identify the mode of delivery. Preferably use “web-based” and/or “mobile” and/or “electronic game” in the title. Avoid ambiguous terms like “online”, “virtual”, “interactive”. Use “Internet-based” only if Intervention includes non-web-based Internet components (e.g. email), use “computer-based” or “electronic” only if offline products are used. Use “virtual” only in the context of “virtual reality” (3-D worlds). Use “online” only in the context of “online support groups”. Complement or substitute product names with broader terms for the class of products (such as “mobile” or “smart phone” instead of “iphone”), especially if the application runs on different platforms.

	1	2	3	4	5	
subitem not at all important	( )	( )	(●)	( )	( )	essential

Clear selection

Does your paper address subitem 1a-i? *

Copy and paste relevant sections from manuscript title (include quotes in quotation marks "like this" to indicate direct quotes from your manuscript), or elaborate on this item by providing additional information not in the ms, or briefly explain why the item is not applicable/relevant for your study

"Recent research has focused on online CBT (including internet-delivered or telephone-based interventions) as a means of delivering cost-effective treatment to populations that are not able to access high-quality psychotherapy services provided in traditional in-office environments. The structured and goal-directed modules of CBT are well-suited to online delivery, and numerous randomized controlled trials (RCTs) have indicated that internet-delivered CBT is highly effective in symptom reduction and depression remission7-9. In recent years, CBT has been integrated with mindfulness meditation following strong evidence for the increased efficacy when CBT and mindfulness meditation are combined10. While other trials support online CBT and mindfulness efficacy vs. wait list controls11-20, no prior trial has compared the efficacy and the cost-effectiveness of online group CBT-M vs. standard office-based group CBT-M."

"The present study has 3 aims: (1) to evaluate whether online group CBT-M plus standard psychiatric care is non-inferior in efficacy to office-based group CBT-M plus standard care as measured by Beck Depression Inventory-II (BDI-II) score in the treatment of adults with major depressive disorder (MDD); (2) to evaluate whether online group CBT-M is more cost-effective than office-based group CBT-M at post-intervention and 6 months post-intervention follow-up; (3) to assess, within the online group CBT-M intervention group, whether digitally recorded adherence data (i.e. online workbooks completed, Fitbit tracked steps, online text messages exchanged, phone sessions completed) predict outcome benefits as indicated by changed BDI-II score."

"Online Intervention: The online group CBT-M program combines software-based workbooks with phone-based Navigator-Coaching that coordinates software interactions (e.g. secure text messaging, Fitbit tracked walking, food monitoring via photography). Each participant is loaned a Fitbit-HR Charge 3, which assesses physical steps and 24-hour heart rate, averaged in 5 second intervals. Intervention content builds on two online RCTs with students6-9, and an RCT with adults with Type 2 Diabetes11-13 (where significant mental health and blood glucose benefits resulted). The content contains 24 workbook chapters on multiple topics derived from focus group study (e.g. Living By Your Truths, Overcoming Wired-ness and Tired-ness, Mindfulness and Relationships, Loss and Grief, and Resilience, Befriending Ourselves, Befriending Your Body with Exercise, Body Image, Intimacy, Forgiveness, Overcoming Procrastination, Dealing with Negative Moods, Stress Resilience, Reducing Performance Anxiety, Cultivating Inspiration), addressed in sequences mutually agreed on by participants and Navigator-Coaches.

The online CBT-M groups address structured tasks that elevate mood and decrease anxiety. Depressive social withdrawals are identified, and reactive withdrawals reflected on while corrective emotional experiences are elicited, structured and supported. Healthy internet interactions are reinforced by the online program content (24 workbooks, 56 videos), with priorities guided by participant interests. While internet-based contacts, generally, can offer distracting 'escapes' (e.g. Netflix movies, video game playing), they can also be productive, [illegible]

socially engaging events (e.g. TED Talks, CBT discussion, mindfulness instructions) where role-modeling coordinates with learning. Group members stimulate and reinforce health practice adoptions (exercise, CBT, mindfulness, sleep hygiene, diet, text-message encouragement) and transformations from distractive media fixations to the positive learning and enjoyment. Online contacts integrate with face-to-face professional and familial contacts, and social intimacy development. Group online CBT-M tasks emphasize applying CBT-mindfulness methods in accord with content themes: de-catastrophizing; overcoming perfectionism; sound sleep for increased vitality; self befriending; courage development; forgiveness; interpersonal mindfulness; and autonomous generosity. This progression coordinates physical and cognitive changes that integrate interpersonal contacts with self-management. The tracking of walking exercise and heart rate (HR) on 24-hour basis captures HR elevations that reflect episodes of anxiety and negative affect (elevated HR with minimal movement). There is selective sharing of Fitbit bio-behavioural data with designated staff and group members. Reduced HR often accompanies mindfulness practice while mildly elevated HR is closely associated with walking (i.e., movement-affected HR). Self-modification goals involve improving autonomic nervous system balance through appropriate exercise, improved sleep hygiene and restorative sleep41."

"

1a-ii) Non-web-based components or important co-interventions in title

Mention non-web-based components or important co-interventions in title, if any (e.g., “with telephone support”).

	1	2	3	4	5	
subitem not at all important	◉	○	○	○	○	essential

Clear selection

Does your paper address subitem 1a-ii?

Copy and paste relevant sections from manuscript title (include quotes in quotation marks "like this" to indicate direct quotes from your manuscript), or elaborate on this item by providing additional information not in the ms, or briefly explain why the item is not applicable/relevant for your study

The non-web-based CBT component is delivered in the standard, empirically supported "person-to-person" format and does not require elaboration.

1a-iii) Primary condition or target group in the title

Mention primary condition or target group in the title, if any (e.g., “for children with Type I Diabetes”) Example: A Web-based and Mobile Intervention with Telephone Support for Children with Type I Diabetes: Randomized Controlled Trial

	1	2	3	4	5	
subitem not at all important	○	○	○	○	◉	essential

Clear selection

Does your paper address subitem 1a-iii? *

Copy and paste relevant sections from manuscript title (include quotes in quotation marks "like this" to indicate direct quotes from your manuscript), or elaborate on this item by providing additional information not in the ms, or briefly explain why the item is not applicable/relevant for your study

The title clearly indicates the primary condition, "Major Depressive Disorder".

1b) ABSTRACT: Structured summary of trial design, methods, results, and conclusions

NPT extension: Description of experimental treatment, comparator, care providers, centers, and blinding status.

1b-i) Key features/functionalities/components of the intervention and comparator in the METHODS section of the ABSTRACT

Mention key features/functionalities/components of the intervention and comparator in the abstract. If possible, also mention theories and principles used for designing the site. Keep in mind the needs of systematic reviewers and indexers by including important synonyms. (Note: Only report in the abstract what the main paper is reporting. If this information is missing from the main body of text, consider adding it)

	1	2	3	4	5	
subitem not at all important	○	○	○	○	◉	essential

Clear selection

Does your paper address subitem 1b-i? *

Copy and paste relevant sections from the manuscript abstract (include quotes in quotation marks "like this" to indicate direct quotes from your manuscript), or elaborate on this item by providing additional information not in the ms, or briefly explain why the item is not applicable/relevant for your study

"All participants receive standard psychiatric care (1 pharmacotherapy focused visit/month with a psychiatrist of 15-30 minute duration). Experimental participants additionally receive online group CBT-M or while control participants receive standard care in-office group CBT-M. The online group CBT-M program (in collaboration with NexJ Health, Inc.) combines exposure to smartphone and computer accessed workbooks with phone-based mental health counselling (16 hours in 16 weeks) that coordinates with ongoing software interactions (e.g. secure text messaging, Fitbit tracked walking). Each participant is loaned a Fitbit-HR Charge 3 to assess physical activity as measured by daily step count. "

1b-ii) Level of human involvement in the METHODS section of the ABSTRACT

Clarify the level of human involvement in the abstract, e.g., use phrases like “fully automated” vs. “therapist/nurse/care provider/physician-assisted” (mention number and expertise of providers involved, if any). (Note: Only report in the abstract what the main paper is reporting. If this information is missing from the main body of text, consider adding it)

	1	2	3	4	5	
subitem not at all important	○	○	○	○	◉	essential

Clear selection

Does your paper address subitem 1b-ii?

Copy and paste relevant sections from the manuscript abstract (include quotes in quotation marks "like this" to indicate direct quotes from your manuscript), or elaborate on this item by providing additional information not in the ms, or briefly explain why the item is not applicable/relevant for your study

Intervention modality is clearly explained in the abstract; all interventions are human-to-human.

1b-iii) Open vs. closed, web-based (self-assessment) vs. face-to-face assessments in the METHODS section of the ABSTRACT

Mention how participants were recruited (online vs. offline), e.g., from an open access website or from a clinic or a closed online user group (closed usergroup trial), and clarify if this was a purely web-based trial, or there were face-to-face components (as part of the intervention or for assessment). Clearly say if outcomes were self-assessed through questionnaires (as common in web-based trials). Note: In traditional offline trials, an open trial (open-label trial) is a type of clinical trial in which both the researchers and participants know which treatment is being administered. To avoid confusion, use “blinded” or “unblinded” to indicated the level of blinding instead of “open”, as “open” in web-based trials usually refers to “open access” (i.e. participants can self-enrol). (Note: Only report in the abstract what the main paper is reporting. If this information is missing from the main body of text, consider adding it)

	1	2	3	4	5	
subitem not at all important	○	○	○	○	◉	essential

Clear selection

Does your paper address subitem 1b-iii?

Copy and paste relevant sections from the manuscript abstract (include quotes in quotation marks "like this" to indicate direct quotes from your manuscript), or elaborate on this item by providing additional information not in the ms, or briefly explain why the item is not applicable/relevant for your study

The assessment format is clearly explained; self-report assessments are delivered through REDCap and the HDRS-24 is administered by a trained, blinded assessor-rater.

1b-iv) RESULTS section in abstract must contain use data

Report number of participants enrolled/assessed in each group, the use/uptake of the intervention (e.g., attrition/adherence metrics, use over time, number of logins etc.), in addition to primary/secondary outcomes. (Note: Only report in the abstract what the main paper is reporting. If this information is missing from the main body of text, consider adding it)

	1	2	3	4	5	
subitem not at all important	◉	○	○	○	○	essential

Clear selection

Does your paper address subitem 1b-iv?

Copy and paste relevant sections from the manuscript abstract (include quotes in quotation marks "like this" to indicate direct quotes from your manuscript), or elaborate on this item by providing additional information not in the ms, or briefly explain why the item is not applicable/relevant for your study

This is a protocol paper and recruitment has not yet taken place. Target participant enrolment numbers are included.

1b-v) CONCLUSIONS/DISCUSSION in abstract for negative trials

Conclusions/Discussions in abstract for negative trials: Discuss the primary outcome - if the trial is negative (primary outcome not changed), and the intervention was not used, discuss whether negative results are attributable to lack of uptake and discuss reasons. (Note: Only report in the abstract what the main paper is reporting. If this information is missing from the main body of text, consider adding it)

	1	2	3	4	5	
subitem not at all important	◉	○	○	○	○	essential

Clear selection

Does your paper address subitem 1b-v?

Copy and paste relevant sections from the manuscript abstract (include quotes in quotation marks "like this" to indicate direct quotes from your manuscript), or elaborate on this item by providing additional information not in the ms, or briefly explain why the item is not applicable/relevant for your study

This paper is a protocol paper and thus there are no results provided in the conclusion/discussion.

INTRODUCTION

2a) In INTRODUCTION: Scientific background and explanation of rationale

2a-i) Problem and the type of system/solution

Describe the problem and the type of system/solution that is object of the study: intended as stand-alone intervention vs. incorporated in broader health care program? Intended for a particular patient population? Goals of the intervention, e.g., being more cost-effective to other interventions, replace or complement other solutions? (Note: Details about the intervention are provided in “Methods” under 5)

	1	2	3	4	5	
subitem not at all important	○	○	○	○	◉	essential

Clear selection

Does your paper address subitem 2a-i? *

Copy and paste relevant sections from the manuscript (include quotes in quotation marks "like this" to indicate direct quotes from your manuscript), or elaborate on this item by providing additional information not in the ms, or briefly explain why the item is not applicable/relevant for your study

"Mental health disorders are Canada's most costly chronic health problem for both health systems and patients, and they are increasing in incidence. The current economic costs of mental health are estimated at $51 billion annually, with $42.3 billion in direct costs to the Canadian healthcare system1. Depression is a commonly diagnosed mental health disorder, representing the most prevalent cause of disability worldwide2. Cognitive Behavioural Therapy (CBT) is the best validated psychotherapy for the treatment of depression, with decades of research demonstrating its efficacy3. Despite its demonstrated efficacy, many individuals are not able to access adequate CBT treatment due to the limitations of face-to-face delivery. Improving access to CBT services is crucial to overcome the treatment barriers— including geographic distance, cost, time, and perceived stigma—that currently prevent access to necessary mental health care for individuals with depression4-6. Recent research has focused on online CBT (including internet-delivered or telephone-based interventions) as a means of delivering cost-effective treatment to populations that are not able to access high-quality psychotherapy services provided in traditional in-office environments. The structured and goal-directed modules of CBT are well-suited to online delivery, and numerous randomized controlled trials (RCTs) have indicated that internet-delivered CBT is highly effective in symptom reduction and depression remission7-9. In recent years, CBT has been integrated with mindfulness meditation following strong evidence for the increased efficacy when CBT and mindfulness meditation are combined10. While other trials support online CBT and mindfulness efficacy vs. wait list controls11-20, no prior trial has compared the efficacy and the cost-effectiveness of online group CBT-M vs. standard office-based group CBT-M.

Previous non-inferiority RCTs have indicated that guided online CBT can be at least as effective as in-person CBT for the treatment of depression, but these studies are limited by the use of small, non-clinical samples and do not include cost-effectiveness comparisons 21-23. Research is now required with a large and adequate sample size and in-depth cost comparisons to fully establish non-inferiority and cost-effectiveness."

2a-ii) Scientific background, rationale: What is known about the (type of) system

Scientific background, rationale: What is known about the (type of) system that is the object of the study (be sure to discuss the use of similar systems for other conditions/diagnoses, if appropiate), motivation for the study, i.e. what are the reasons for and what is the context for this specific study, from which stakeholder viewpoint is the study performed, potential impact of findings [2]. Briefly justify the choice of the comparator.

	1	2	3	4	5	
subitem not at all important	○	○	○	○	◉	essential

Clear selection

Does your paper address subitem 2a-ii? *

Copy and paste relevant sections from the manuscript (include quotes in quotation marks "like this" to indicate direct quotes from your manuscript), or elaborate on this item by providing additional information not in the ms, or briefly explain why the item is not applicable/relevant for your study

"Mental health disorders are Canada's most costly chronic health problem for both health systems and patients, and they are increasing in incidence. The current economic costs of mental health are estimated at $51 billion annually, with $42.3 billion in direct costs to the Canadian healthcare system1. Depression is a commonly diagnosed mental health disorder, representing the most prevalent cause of disability worldwide2. Cognitive Behavioural Therapy (CBT) is the best validated psychotherapy for the treatment of depression, with decades of research demonstrating its efficacy3. Despite its demonstrated efficacy, many individuals are not able to access adequate CBT treatment due to the limitations of face-to-face delivery. Improving access to CBT services is crucial to overcome the treatment barriers— including geographic distance, cost, time, and perceived stigma—that currently prevent access to necessary mental health care for individuals with depression4-6. Recent research has focused on online CBT (including internet-delivered or telephone-based interventions) as a means of delivering cost-effective treatment to populations that are not able to access high-quality psychotherapy services provided in traditional in-office environments. The structured and goal-directed modules of CBT are well-suited to online delivery, and numerous randomized controlled trials (RCTs) have indicated that internet-delivered CBT is highly effective in symptom reduction and depression remission7-9. In recent years, CBT has been integrated with mindfulness meditation following strong evidence for the increased efficacy when CBT and mindfulness meditation are combined10. While other trials support online CBT and mindfulness efficacy vs. wait list controls11-20, no prior trial has compared the efficacy and the cost-effectiveness of online group CBT-M vs. standard office-based group CBT-M.

Previous non-inferiority RCTs have indicated that guided online CBT can be at least as effective as in-person CBT for the treatment of depression, but these studies are limited by the use of small, non-clinical samples and do not include cost-effectiveness comparisons 21-23. Research is now required with a large and adequate sample size and in-depth cost comparisons to fully establish non-inferiority and cost-effectiveness."

2b) In INTRODUCTION: Specific objectives or hypotheses

Does your paper address CONSORT subitem 2b? *

Copy and paste relevant sections from the manuscript (include quotes in quotation marks "like this" to indicate direct quotes from your manuscript), or elaborate on this item by providing additional information not in the ms, or briefly explain why the item is not applicable/relevant for your study

"Online group CBT-M will be non-inferior to standard, office-based group CBT in the treatment of MDD (as indicated by BDI-II score change) when online and office-based treatment groups are compared, using both intention-to-treat (ITT) and per protocol analyses (PP). Online group CBT-MM will be more cost-effective than standard, office-based group CBT, as measured by cumulative costs and quality-adjusted life-years (QALYs) calculated in the cost utility analysis. Within the online group CBT-M intervention participant group, digitally recorded adherence data will predict outcome benefits (BDI-II change). Adherence over time will be compared within each participant using a generalized estimating equation (GEE) logistic regression model with an AR1 correlation structure."

METHODS

3a) Description of trial design (such as parallel, factorial) including allocation ratio

Does your paper address CONSORT subitem 3a? *

Copy and paste relevant sections from the manuscript (include quotes in quotation marks "like this" to indicate direct quotes from your manuscript), or elaborate on this item by providing additional information not in the ms, or briefly explain why the item is not applicable/relevant for your study

"Participants will be identified from wait-lists within Centre for Addiction and Mental Health (CAMH General Adult Psychiatry and Health Systems Division which services thousands of MDD patients annually. Participants may also be identified though contacts with other Toronto outpatient clinics. Potentially eligible participants will be identified by a Research Coordinator who will explore interest with the participant, and if interest is expressed, the Research Coordinator will review and explain the study. Eligibility screening and written consent will be undertaken in person prior to randomization. The study biostatistician (GT) will perform electronic randomization with study IDs blindly assigned to intervention (online group CBT-M plus standard psychiatric care) and control (office-based group CBT-M plus standard psychiatric care) groups. The Study ID information with the respective group allocation will be transferred onto cards placed in opaque, individual, sealed envelopes. After a participant completes baseline questionnaires, the Research Coordinator will open the next envelope in sequence to determine group allocation and Study ID. Based off of the results of previous successful RCTs including our own conducted at the same study site, we will recruit 100 participants per group or 200 overall (total N = 200). One-sided type 1 error rate is set at 5%. We chose a margin of 3 and a correlation of 0.70, giving a sample size of 78 per group in 2 groups, allowing for a drop-out rate of 20%. This sample is more than adequate for detection of small to medium effect sizes."

3b) Important changes to methods after trial commencement (such as eligibility criteria), with reasons

Does your paper address CONSORT subitem 3b? *

Copy and paste relevant sections from the manuscript (include quotes in quotation marks "like this" to indicate direct quotes from your manuscript), or elaborate on this item by providing additional information not in the ms, or briefly explain why the item is not applicable/relevant for your study

No, as this is a protocol paper and the trial has not yet started.

3b-i) Bug fixes, Downtimes, Content Changes

Bug fixes, Downtimes, Content Changes: ehealth systems are often dynamic systems. A description of changes to methods therefore also includes important changes made on the intervention or comparator during the trial (e.g., major bug fixes or changes in the functionality or content) (5-iii) and other "unexpected events" that may have influenced study design such as staff changes, system failures/downtimes, etc. [2].

	1	2	3	4	5	
subitem not at all important	(●)	( )	( )	( )	( )	essential

Clear selection

Does your paper address subitem 3b-i?

Copy and paste relevant sections from the manuscript (include quotes in quotation marks "like this" to indicate direct quotes from your manuscript), or elaborate on this item by providing additional information not in the ms, or briefly explain why the item is not applicable/relevant for your study

Your answer

4a) Eligibility criteria for participants

Does your paper address CONSORT subitem 4a? *

Copy and paste relevant sections from the manuscript (include quotes in quotation marks "like this" to indicate direct quotes from your manuscript), or elaborate on this item by providing additional information not in the ms, or briefly explain why the item is not applicable/relevant for your study

"Inclusion Criteria
The inclusion criteria are: (1) individuals 18-60 years of age; (2) Beck Depression Inventory-II of at least mild severity (BDI-II score ≥ 14) with no upper severity limit37; (3) MINI-confirmed diagnosis of MDD38; (4) fluent in English. All participants are diagnosed by a CAMH staff psychiatrist with diagnosis confirmed via MINI International Neuropsychiatric Interview (MINI)38, administered at the screening visit.

Exclusion Criteria
The exclusion criteria are: (1) individuals currently receiving weekly structured psychotherapy; (2) individuals who meet DSM-V criteria for severe alcohol/substance use disorder (in the past 3 months), borderline personality disorder, schizophrenia or any other primary psychotic disorder, bipolar disorder or obsessive-compulsive disorder; (3) individuals who manifest clinically significant suicidal ideation defined as imminent intent or attempted suicide (in the past 6 months); (4) individuals who are judged to have treatment resistant depression (TRD), as defined by failure in at least two trials of antidepressant medications and/or a course of psychotherapy during the current depressive episode38-40."

4a-i) Computer / Internet literacy

Computer / Internet literacy is often an implicit “de facto” eligibility criterion - this should be explicitly clarified.

	1	2	3	4	5	
subitem not at all important	(●)	( )	( )	( )	( )	essential

Clear selection

Does your paper address subitem 4a-i?

Copy and paste relevant sections from the manuscript (include quotes in quotation marks "like this" to indicate direct quotes from your manuscript), or elaborate on this item by providing additional information not in the ms, or briefly explain why the item is not applicable/relevant for your study

Your answer

4a-ii) Open vs. closed, web-based vs. face-to-face assessments:

Open vs. closed, web-based vs. face-to-face assessments: Mention how participants were recruited (online vs. offline), e.g., from an open access website or from a clinic, and clarify if this was a purely web-based trial, or there were face-to-face components (as part of the intervention or for assessment), i.e., to what degree got the study team to know the participant. In online-only trials, clarify if participants were quasi-anonymous and whether having multiple identities was possible or whether technical or logistical measures (e.g., cookies, email confirmation, phone calls) were used to detect/prevent these.

	1	2	3	4	5	
subitem not at all important	○	○	○	○	◉	essential

Clear selection

Does your paper address subitem 4a-ii? *

Copy and paste relevant sections from the manuscript (include quotes in quotation marks "like this" to indicate direct quotes from your manuscript), or elaborate on this item by providing additional information not in the ms, or briefly explain why the item is not applicable/relevant for your study

"Participants will be identified from wait-lists within Centre for Addiction and Mental Health (CAMH General Adult Psychiatry and Health Systems Division which services thousands of MDD patients annually. Participants may also be identified though contacts with other Toronto outpatient clinics. Potentially eligible participants will be identified by a Research Coordinator who will explore interest with the participant, and if interest is expressed, the Research Coordinator will review and explain the study. Eligibility screening and written consent will be undertaken in person prior to randomization. The study biostatistician (GT) will perform electronic randomization with study IDs blindly assigned to intervention (online group CBT-M plus standard psychiatric care) and control (office-based group CBT-M plus standard psychiatric care) groups. The Study ID information with the respective group allocation will be transferred onto cards placed in opaque, individual, sealed envelopes. After a participant completes baseline questionnaires, the Research Coordinator will open the next envelope in sequence to determine group allocation and Study ID. Based off of the results of previous successful RCTs including our own conducted at the same study site, we will recruit 100 participants per group or 200 overall (total N = 200). One-sided type 1 error rate is set at 5%. We chose a margin of 3 and a correlation of 0.70, giving a sample size of 78 per group in 2 groups, allowing for a drop-out rate of 20%. This sample is more than adequate for detection of small to medium effect sizes."

4a-iii) Information giving during recruitment

Information given during recruitment. Specify how participants were briefed for recruitment and in the informed consent procedures (e.g., publish the informed consent documentation as appendix, see also item X26), as this information may have an effect on user self-selection, user expectation and may also bias results.

	1	2	3	4	5	
subitem not at all important	(•)	( )	( )	( )	( )	essential

Clear selection

Does your paper address subitem 4a-iii?

Copy and paste relevant sections from the manuscript (include quotes in quotation marks "like this" to indicate direct quotes from your manuscript), or elaborate on this item by providing additional information not in the ms, or briefly explain why the item is not applicable/relevant for your study

Patients who are deemed suitable to participate in research will be asked by their Attending Physician/Clinician for verbal consent to receive more information about the research project. The first point of contact for flagged patients will be their Attending Physician/Clinician. The patient's Attending Physician/Clinician will obtain verbal consent to contact the them with more information about the Research Project. If the patient declines, the CLEARR Coordinator will document the REB number and that the participant did not consent to receiving further information about the research study. The patients who provide verbal consent will be introduced to the Research Coordinator (who will also be the CLEARR Coordinator) to receive more information about the Research Project. If the patient provides verbal consent to be contacted about the Research Project, they will be approached by the CLEARR Coordinator to receive more information about the Research Project. Prior to obtaining informed consent, no Authorized Research Personnel other than the CLEARR Coordinator will have access to additional PHI. The Research Coordinator will provide further information and if the patient is interested in potentially participating, a screening visit will be scheduled. Prior to any screening taking place, the Research Coordinator will receive written consent from the participant. Participants will sign read the attached information sheet and discuss any questions with the Research Coordinator, then sign informed consent form (provided in this application).

COVID-19 Amendment to Consent

For the duration of the time that CAMH is limiting on-site non-essential research visits, consent for study participation will be obtained remotely. The guidelines informing the remote consent process are CAMH SOP GR 106, the CAMH Ethical Considerations for Remote Consent document, the CAMH Research Guidelines for Virtual Participant Sessions, and the CAMH SOP HSR 201 – Documentation Requirements for Human Subject Research.

The steps to obtaining remote consent that will be followed are as follows:

1. Participant is provided with an overview of the study, and options for receiving the informed consent form (ICF) and conducting the consent discussion

Prospective participants will be contacted using the Initial Telephone Contact Script provided by REB and tailored to reflect the specific details of this study. The telephone script that will be used is attached to this REB Amendment (document titled Initial Telephone Contact Script - Gratzer REB #087/2020). Prior to the consent discussion, participants will be provided with the REB-approved ICF to assist in the consent discussion. The updated ICF that reflects the COVID-19 REB Amendments being made is attached to this REB Amendment application. The ICF will be provided to participants via their preferred method for receiving the consent material (email or by mail). Participants will give verbal consent to receive the ICF via mail or email in the initial telephone call made to participants by the [illegible]

Research Coordinator. A second appointment will be made based on participant availability to discuss the ICF, ensuring adequate time for the participant to receive the ICF and read it over in detail. Participants will be provided with as much time as they need to review the consent form both prior to and during the appointment when consent is obtained.

2. Consent discussion will occur via CAMH-approved teleconference (via Webex) or telephone

The consent discussion will occur via teleconference, using Webex, or telephone if the participant is not comfortable using Webex. If the participant consents to receiving information regarding Webex over email, an email will be sent to participants with a meeting invite using the email template included in this REB Amendment application (document titled Email Template for Participant Contact - Gratzer REB #087/2020). Remote visits, including the consent visit, will occur in accordance with the CAH Research Guidelines for Virtual Participant Sessions. Research participant identification will occur at the beginning of the consent visit via showing the research personnel conducting the visit government-issued photo ID (via Webex) during the first encounter with the research personnel. If the consent visit is occurring over the phone at the participants preference, participant identity will be confirmed using double identifiers (full name and date of birth). Research personnel will document their verification of research participant identity in a paper copy of the Participant Contact Information and Identification Form (provided in CAMH SOP GR 106), which will be transferred onto CAMH property by research personnel as feasible. All participant questions will be answered and time will be taken to fully address the entirety of the ICF as would be done if the consent process was occurring in person. The research personnel conducting the consent visit will confirm that the individual has the ICF with them and can follow along during the discussion, pausing during the consent discussion to ask if the prospective participant wants to further discuss or review any information, answer questions throughout the consent discussion to gauge engagement and comprehension, and to support participants in their use of technological platforms including explaining how participants can complete the consent process in lay terms.

3. Informed consent is documented

Informed consent will be documented, as per the CAMH guidelines on Ethical Considerations for Remote Consent. Research personnel will confirm that the participant has the technical capability to print the ICF in order to sign it. Participants will sign the ICF using wet ink and the participant will send a scanned copy or photograph of the signed ICF back to the research personnel via email. When the ICF is received by research personnel, it will be signed by the team member conducting the consent discussion and dated. Participants will be sent a pre-paid envelope to return the original hard-copy ICF to CAMH. It will be documented on the paper copy of the Participant Contact Information and Identification Form that consent has been obtained.

4. Participant is provided with completed, fully signed ICF

Participants will be provided with a completed, fully signed ICF via email or regular mail, depending on their preference. Paper Participant Records will document that the participant has been provided with the fully signed ICF and these records will be transported to CAMH when on-site activity resumes as per SOP GR 106.

4b) Settings and locations where the data were collected

Does your paper address CONSORT subitem 4b? *

Copy and paste relevant sections from the manuscript (include quotes in quotation marks "like this" to indicate direct quotes from your manuscript), or elaborate on this item by providing additional information not in the ms, or briefly explain why the item is not applicable/relevant for your study

All data is collected from participants in Toronto, Ontario, Canada.

4b-i) Report if outcomes were (self-)assessed through online questionnaires

Clearly report if outcomes were (self-)assessed through online questionnaires (as common in web-based trials) or otherwise.

	1	2	3	4	5	
subitem not at all important	○	○	○	○	◉	essential

Clear selection

Does your paper address subitem 4b-i? *

Copy and paste relevant sections from the manuscript (include quotes in quotation marks "like this" to indicate direct quotes from your manuscript), or elaborate on this item by providing additional information not in the ms, or briefly explain why the item is not applicable/relevant for your study

Self-report assessments are administered to participants via REDCap.

4b-ii) Report how institutional affiliations are displayed

Report how institutional affiliations are displayed to potential participants [on ehealth media], as affiliations with prestigious hospitals or universities may affect volunteer rates, use, and reactions with regards to an intervention.(Not a required item – describe only if this may bias results)

	1	2	3	4	5	
subitem not at all important	◉	○	○	○	○	essential

Clear selection

Does your paper address subitem 4b-ii?

Copy and paste relevant sections from the manuscript (include quotes in quotation marks "like this" to indicate direct quotes from your manuscript), or elaborate on this item by providing additional information not in the ms, or briefly explain why the item is not applicable/relevant for your study

All research team members are affiliated with the Centre for Addiction and Mental Health. No other information is provided on research team affiliations.

5) The interventions for each group with sufficient details to allow replication, including how and when they were actually administered

5-i) Mention names, credential, affiliations of the developers, sponsors, and owners

Mention names, credential, affiliations of the developers, sponsors, and owners [6] (if authors/evaluators are owners or developer of the software, this needs to be declared in a “Conflict of interest” section or mentioned elsewhere in the manuscript).

	1	2	3	4	5	
subitem not at all important	◉	○	○	○	○	essential

Clear selection

Does your paper address subitem 5-i?

Copy and paste relevant sections from the manuscript (include quotes in quotation marks "like this" to indicate direct quotes from your manuscript), or elaborate on this item by providing additional information not in the ms, or briefly explain why the item is not applicable/relevant for your study

The software used was developed by NexJ Health, Inc.

"Licences for and use of the smartphone app, NexJ Connected Wellness (NCW), are provided for this trial free-of-charge by NexJ Health, Inc. (Toronto, Ontario). The platform is cloud-based and hosted on IBM Cloud's Tier 4 data centers in Toronto and Montreal. IBM Cloud maintains an industry-leading array of security and privacy certifications, including SOC 1, 2, and 3, as well as the ISO 27000 family of certifications. By hosting on IBM Cloud, NexJ Health benefits from extremely high system uptime, fault tolerance, and security controls. As part of NexJ's extensive security processes and controls, 24/7 monitoring is conducted, which includes annual third-party penetration testing. All data is encrypted at rest and in transit, and no patient information is stored on the patient's device. The summary Privacy Impact Analysis is provided upon request. Privacy Policy and Terms of Use are also provided on request. See Appendices A1 and A2 for the Privacy Policy and Terms of Use."

5-ii) Describe the history/development process

Describe the history/development process of the application and previous formative evaluations (e.g., focus groups, usability testing), as these will have an impact on adoption/use rates and help with interpreting results.

	1	2	3	4	5	
subitem not at all important	(selected)	○	○	○	○	essential

Clear selection

Does your paper address subitem 5-ii?

Copy and paste relevant sections from the manuscript (include quotes in quotation marks "like this" to indicate direct quotes from your manuscript), or elaborate on this item by providing additional information not in the ms, or briefly explain why the item is not applicable/relevant for your study

Your answer

5-iii) Revisions and updating

Revisions and updating. Clearly mention the date and/or version number of the application/intervention (and comparator, if applicable) evaluated, or describe whether the intervention underwent major changes during the evaluation process, or whether the development and/or content was "frozen" during the trial. Describe dynamic components such as news feeds or changing content which may have an impact on the replicability of the intervention (for unexpected events see item 3b).

	1	2	3	4	5	
subitem not at all important	(•)	( )	( )	( )	( )	essential

Clear selection

Does your paper address subitem 5-iii?

Copy and paste relevant sections from the manuscript (include quotes in quotation marks "like this" to indicate direct quotes from your manuscript), or elaborate on this item by providing additional information not in the ms, or briefly explain why the item is not applicable/relevant for your study

There have been no revisions of the intervention.

5-iv) Quality assurance methods

Provide information on quality assurance methods to ensure accuracy and quality of information provided [1], if applicable.

	1	2	3	4	5	
subitem not at all important	(•)	( )	( )	( )	( )	essential

Clear selection

Does your paper address subitem 5-iv?

Copy and paste relevant sections from the manuscript (include quotes in quotation marks "like this" to indicate direct quotes from your manuscript), or elaborate on this item by providing additional information not in the ms, or briefly explain why the item is not applicable/relevant for your study

Your answer

5-v) Ensure replicability by publishing the source code, and/or providing screenshots/screen-capture video, and/or providing flowcharts of the algorithms used

Ensure replicability by publishing the source code, and/or providing screenshots/screen-capture video, and/or providing flowcharts of the algorithms used. Replicability (i.e., other researchers should in principle be able to replicate the study) is a hallmark of scientific reporting.

	1	2	3	4	5	
subitem not at all important	(x)	( )	( )	( )	( )	essential

Clear selection

Does your paper address subitem 5-v?

Copy and paste relevant sections from the manuscript (include quotes in quotation marks "like this" to indicate direct quotes from your manuscript), or elaborate on this item by providing additional information not in the ms, or briefly explain why the item is not applicable/relevant for your study

Your answer

5-vi) Digital preservation

Digital preservation: Provide the URL of the application, but as the intervention is likely to change or disappear over the course of the years; also make sure the intervention is archived (Internet Archive, webcitation.org, and/or publishing the source code or screenshots/videos alongside the article). As pages behind login screens cannot be archived, consider creating demo pages which are accessible without login.

	1	2	3	4	5	
subitem not at all important	◉	○	○	○	○	essential

Clear selection

Does your paper address subitem 5-vi?

Copy and paste relevant sections from the manuscript (include quotes in quotation marks "like this" to indicate direct quotes from your manuscript), or elaborate on this item by providing additional information not in the ms, or briefly explain why the item is not applicable/relevant for your study

Your answer

5-vii) Access

Access: Describe how participants accessed the application, in what setting/context, if they had to pay (or were paid) or not, whether they had to be a member of specific group. If known, describe how participants obtained “access to the platform and Internet” [1]. To ensure access for editors/reviewers/readers, consider to provide a “backdoor” login account or demo mode for reviewers/readers to explore the application (also important for archiving purposes, see vi).

	1	2	3	4	5	
subitem not at all important	◉	○	○	○	○	essential

Clear selection

Does your paper address subitem 5-vii? *

Copy and paste relevant sections from the manuscript (include quotes in quotation marks "like this" to indicate direct quotes from your manuscript), or elaborate on this item by providing additional information not in the ms, or briefly explain why the item is not applicable/relevant for your study

The app is freely accessible via download from the Apple Store or via Google Play.

5-viii) Mode of delivery, features/functionalities/components of the intervention and comparator, and the theoretical framework

Describe mode of delivery, features/functionalities/components of the intervention and comparator, and the theoretical framework [6] used to design them (instructional strategy [1], behaviour change techniques, persuasive features, etc., see e.g., [7, 8] for terminology). This includes an in-depth description of the content (including where it is coming from and who developed it) [1],” whether [and how] it is tailored to individual circumstances and allows users to track their progress and receive feedback” [6]. This also includes a description of communication delivery channels and – if computer-mediated communication is a component – whether communication was synchronous or asynchronous [6]. It also includes information on presentation strategies [1], including page design principles, average amount of text on pages, presence of hyperlinks to other resources, etc. [1].

	1	2	3	4	5	
subitem not at all important	○	○	○	○	◉	essential

Clear selection

Does your paper address subitem 5-viii? *

Copy and paste relevant sections from the manuscript (include quotes in quotation marks "like this" to indicate direct quotes from your manuscript), or elaborate on this item by providing additional information not in the ms, or briefly explain why the item is not applicable/relevant for your study

"Interventions
Online Intervention: The online group CBT-M program combines software-based workbooks with phone-based Navigator-Coaching that coordinates software interactions (e.g. secure text messaging, Fitbit tracked walking, food monitoring via photography). Each participant is loaned a Fitbit-HR Charge 3, which assesses physical steps and 24-hour heart rate, averaged in 5 second intervals. Intervention content builds on two online RCTs with students6-9, and an RCT with adults with Type 2 Diabetes11-13 (where significant mental health and blood glucose benefits resulted). The content contains 24 workbook chapters on multiple topics derived from focus group study (e.g. Living By Your Truths, Overcoming Wired-ness and Tired-ness, Mindfulness and Relationships, Loss and Grief, and Resilience, Befriending Ourselves, Befriending Your Body with Exercise, Body Image, Intimacy, Forgiveness, Overcoming Procrastination, Dealing with Negative Moods, Stress Resilience, Reducing Performance Anxiety, Cultivating Inspiration), addressed in sequences mutually agreed on by participants and Navigator-Coaches.

The online CBT-M groups address structured tasks that elevate mood and decrease anxiety. Depressive social withdrawals are identified, and reactive withdrawals reflected on while corrective emotional experiences are elicited, structured and supported. Healthy internet interactions are reinforced by the online program content (24 workbooks, 56 videos), with priorities guided by participant interests. While internet-based contacts, generally, can offer distracting 'escapes' (e.g. Netflix movies, video game playing), they can also be productive, socially engaging events (e.g. TED Talks, CBT discussion, mindfulness instructions) where role-modeling coordinates with learning. Group members stimulate and reinforce health practice adoptions (exercise, CBT, mindfulness, sleep hygiene, diet, text-message encouragement) and transformations from distractive media fixations to the positive learning and enjoyment. Online contacts integrate with face-to-face professional and familial contacts, and social intimacy development. Group online CBT-M tasks emphasize applying CBT-mindfulness methods in accord with content themes: de-catastrophizing; overcoming perfectionism; sound sleep for increased vitality; self befriending; courage development; forgiveness; interpersonal mindfulness; and autonomous generosity. This progression coordinates physical and cognitive changes that integrate interpersonal contacts with self-management. The tracking of walking exercise and heart rate (HR) on 24-hour basis captures HR elevations that reflect episodes of anxiety and negative affect (elevated HR with minimal movement). There is selective sharing of Fitbit bio-behavioural data with designated staff and group members. Reduced HR often accompanies mindfulness practice while mildly elevated HR is closely associated with walking (i.e., movement-affected HR). Self-modification goals involve improving autonomic nervous system balance through appropriate exercise, improved sleep hygiene and restorative sleep41.

Office-Based Intervention: the on-site, usual-care CBT group follows the structure of the Mind Over Mood workbook (MOM, Guilford Press) in reviewing CBT concepts and [illegible]

procedures. A series of work sheets assist participants in differentiating moods, and in differentiating moods from thoughts and situational influences, leading to modifications of thinking, behaviour, emotion and mood.

Difficulties with cognitive change are assumed to be associated with self-acceptance deficits and negative mood dominance. Increased self-acceptance is linked with mindfulness experiences, which involve 'more focusing on the present moment' and on non-judgmental acceptance of ongoing experience. Mindful breathing awareness is described in content on 'balanced deep breathing' and in tension releases achieved via Progressive Muscle Relaxation practice.

An emphasized structure is the automatic thought record (ATM) by which negative, disturbing thoughts are identified, and attention directed to alternate thoughts judged clearer and less distorted. These transitions are more difficult when immersions in negative moods diminish the confidence in finding a 'better' thought. Accordingly, self-efficacy is an important goal, emphasized in exchanges of support between group members and in enhanced experiences of self-acceptance.

Behavioural activations (BA) are structured for the development of increased awareness and observations of how self-control resources elicit gratifying activities. On the basis of BA practices, cognitive restructuring (CR) strategies utilize the ATM or experiential approximations."

5-ix) Describe use parameters

Describe use parameters (e.g., intended “doses” and optimal timing for use). Clarify what instructions or recommendations were given to the user, e.g., regarding timing, frequency, heaviness of use, if any, or was the intervention used ad libitum.

	1	2	3	4	5	
subitem not at all important	◉	○	○	○	○	essential

Clear selection

Does your paper address subitem 5-ix?

Copy and paste relevant sections from the manuscript (include quotes in quotation marks "like this" to indicate direct quotes from your manuscript), or elaborate on this item by providing additional information not in the ms, or briefly explain why the item is not applicable/relevant for your study

Your answer

5-x) Clarify the level of human involvement

Clarify the level of human involvement (care providers or health professionals, also technical assistance) in the e-intervention or as co-intervention (detail number and expertise of professionals involved, if any, as well as “type of assistance offered, the timing and frequency of the support, how it is initiated, and the medium by which the assistance is delivered”. It may be necessary to distinguish between the level of human involvement required for the trial, and the level of human involvement required for a routine application outside of a RCT setting (discuss under item 21 – generalizability).

	1	2	3	4	5	
subitem not at all important	○	○	○	○	◉	essential

Clear selection

Does your paper address subitem 5-x?

Copy and paste relevant sections from the manuscript (include quotes in quotation marks "like this" to indicate direct quotes from your manuscript), or elaborate on this item by providing additional information not in the ms, or briefly explain why the item is not applicable/relevant for your study

"Interventions: All participants receive standard psychiatric care (1 pharmacotherapy focused visit/month with a psychiatrist of 15-30 minute duration). Experimental participants additionally receive online group CBT-M or while control participants receive standard care in-office group CBT-M. The online group CBT-M program (in collaboration with NexJ Health, Inc.) combines exposure to smartphone and computer accessed workbooks with phone-based mental health counselling (16 hours in 16 weeks) that coordinates with ongoing software interactions (e.g. secure text messaging, Fitbit tracked walking). Each participant is loaned a Fitbit-HR Charge 3 to assess physical activity as measured by daily step count."

5-xi) Report any prompts/reminders used

Report any prompts/reminders used: Clarify if there were prompts (letters, emails, phone calls, SMS) to use the application, what triggered them, frequency etc. It may be necessary to distinguish between the level of prompts/reminders required for the trial, and the level of prompts/reminders for a routine application outside of a RCT setting (discuss under item 21 – generalizability).

	1	2	3	4	5	
subitem not at all important	◉	○	○	○	○	essential

Clear selection

Does your paper address subitem 5-xi? *

Copy and paste relevant sections from the manuscript (include quotes in quotation marks "like this" to indicate direct quotes from your manuscript), or elaborate on this item by providing additional information not in the ms, or briefly explain why the item is not applicable/relevant for your study

Prompts are not used, and not incorporated into the intervention structure.

5-xii) Describe any co-interventions (incl. training/support)

Describe any co-interventions (incl. training/support): Clearly state any interventions that are provided in addition to the targeted eHealth intervention, as ehealth intervention may not be designed as stand-alone intervention. This includes training sessions and support [1]. It may be necessary to distinguish between the level of training required for the trial, and the level of training for a routine application outside of a RCT setting (discuss under item 21 – generalizability.

	1	2	3	4	5	
subitem not at all important	◉	○	○	○	○	essential

Clear selection

Does your paper address subitem 5-xii? *

Copy and paste relevant sections from the manuscript (include quotes in quotation marks "like this" to indicate direct quotes from your manuscript), or elaborate on this item by providing additional information not in the ms, or briefly explain why the item is not applicable/relevant for your study

There are no co-interventions provided.

6a) Completely defined pre-specified primary and secondary outcome measures, including how and when they were assessed

Does your paper address CONSORT subitem 6a? *

Copy and paste relevant sections from the manuscript (include quotes in quotation marks "like this" to indicate direct quotes from your manuscript), or elaborate on this item by providing additional information not in the ms, or briefly explain why the item is not applicable/relevant for your study

"Measures: Primary outcome: Beck Depression Inventory-II (BDI-II)36; Secondary outcomes: Anxiety (Beck Anxiety Inventory)43, depression (i.e., Quick Inventory of Depressive Symptomatology; (QIDS)44 and the 24-item Hamilton Depression Rating Scale (HDRS-24)45, mindfulness (Five-Facet Mindfulness Questionnaire)46, quality of life (Euro Qol-5D)47,48, patient costs (Health Care Cost Diary for Major Depression)49 and pain (Brief Pain Inventory)50."

6a-i) Online questionnaires: describe if they were validated for online use and apply CHERRIES items to describe how the questionnaires were designed/deployed

If outcomes were obtained through online questionnaires, describe if they were validated for online use and apply CHERRIES items to describe how the questionnaires were designed/deployed [9].

	1	2	3	4	5	
subitem not at all important	(•)	( )	( )	( )	( )	essential

Clear selection

Does your paper address subitem 6a-i?

Copy and paste relevant sections from manuscript text

Your answer

6a-ii) Describe whether and how “use” (including intensity of use/dosage) was defined/measured/monitored

Describe whether and how “use” (including intensity of use/dosage) was defined/measured/monitored (logins, logfile analysis, etc.). Use/adoption metrics are important process outcomes that should be reported in any ehealth trial.

	1	2	3	4	5	
subitem not at all important	(●)	○	○	○	○	essential

Clear selection

Does your paper address subitem 6a-ii?

Copy and paste relevant sections from manuscript text

Your answer

6a-iii) Describe whether, how, and when qualitative feedback from participants was obtained

Describe whether, how, and when qualitative feedback from participants was obtained (e.g., through emails, feedback forms, interviews, focus groups).

	1	2	3	4	5	
subitem not at all important	(●)	○	○	○	○	essential

Clear selection

Does your paper address subitem 6a-iii?

Copy and paste relevant sections from manuscript text

Your answer

6b) Any changes to trial outcomes after the trial commenced, with reasons

Does your paper address CONSORT subitem 6b? *

Copy and paste relevant sections from the manuscript (include quotes in quotation marks "like this" to indicate direct quotes from your manuscript), or elaborate on this item by providing additional information not in the ms, or briefly explain why the item is not applicable/relevant for your study

The trial has not yet commenced.

7a) How sample size was determined

NPT: When applicable, details of whether and how the clustering by care provides or centers was addressed

7a-i) Describe whether and how expected attrition was taken into account when calculating the sample size

Describe whether and how expected attrition was taken into account when calculating the sample size.

	1	2	3	4	5	
subitem not at all important	○	○	○	○	◉	essential

Clear selection

Does your paper address subitem 7a-i?

Copy and paste relevant sections from manuscript title (include quotes in quotation marks "like this" to indicate direct quotes from your manuscript), or elaborate on this item by providing additional information not in the ms, or briefly explain why the item is not applicable/relevant for your study

"Based off of the results of previous successful RCTs including our own conducted at the same study site, we will recruit 100 participants per group or 200 overall (total N = 200). One-sided type 1 error rate is set at 5%. We chose a margin of 3 and a correlation of 0.70, giving a sample size of 78 per group in 2 groups, allowing for a drop-out rate of 20%. This sample is more than adequate for detection of small to medium effect sizes."

7b) When applicable, explanation of any interim analyses and stopping guidelines

Does your paper address CONSORT subitem 7b? *

Copy and paste relevant sections from the manuscript (include quotes in quotation marks "like this" to indicate direct quotes from your manuscript), or elaborate on this item by providing additional information not in the ms, or briefly explain why the item is not applicable/relevant for your study

The study has not started thus this is not applicable.

8a) Method used to generate the random allocation sequence

NPT: When applicable, how care providers were allocated to each trial group

Does your paper address CONSORT subitem 8a? *

Copy and paste relevant sections from the manuscript (include quotes in quotation marks "like this" to indicate direct quotes from your manuscript), or elaborate on this item by providing additional information not in the ms, or briefly explain why the item is not applicable/relevant for your study

The study has not started thus this is not applicable.

8b) Type of randomisation; details of any restriction (such as blocking and block size)

Does your paper address CONSORT subitem 8b? *

Copy and paste relevant sections from the manuscript (include quotes in quotation marks "like this" to indicate direct quotes from your manuscript), or elaborate on this item by providing additional information not in the ms, or briefly explain why the item is not applicable/relevant for your study

"The study biostatistician (GT) will perform electronic randomization with study IDs blindly assigned to intervention (online group CBT-M plus standard psychiatric care) and control (office-based group CBT-M plus standard psychiatric care) groups. The Study ID information with the respective group allocation will be transferred onto cards placed in opaque, individual, sealed envelopes. After a participant completes baseline questionnaires, the Research Coordinator will open the next envelope in sequence to determine group allocation and Study ID."

9) Mechanism used to implement the random allocation sequence (such as sequentially numbered containers), describing any steps taken to conceal the sequence until interventions were assigned

Does your paper address CONSORT subitem 9? *

Copy and paste relevant sections from the manuscript (include quotes in quotation marks "like this" to indicate direct quotes from your manuscript), or elaborate on this item by providing additional information not in the ms, or briefly explain why the item is not applicable/relevant for your study

"The study biostatistician (GT) will perform electronic randomization with study IDs blindly assigned to intervention (online group CBT-M plus standard psychiatric care) and control (office-based group CBT-M plus standard psychiatric care) groups. The Study ID information with the respective group allocation will be transferred onto cards placed in opaque, individual, sealed envelopes. After a participant completes baseline questionnaires, the Research Coordinator will open the next envelope in sequence to determine group allocation and Study ID."

10) Who generated the random allocation sequence, who enrolled participants, and who assigned participants to interventions

Does your paper address CONSORT subitem 10? *

Copy and paste relevant sections from the manuscript (include quotes in quotation marks "like this" to indicate direct quotes from your manuscript), or elaborate on this item by providing additional information not in the ms, or briefly explain why the item is not applicable/relevant for your study

"The study biostatistician (GT) will perform electronic randomization with study IDs blindly assigned to intervention (online group CBT-M plus standard psychiatric care) and control (office-based group CBT-M plus standard psychiatric care) groups. The Study ID information with the respective group allocation will be transferred onto cards placed in opaque, individual, sealed envelopes. After a participant completes baseline questionnaires, the Research Coordinator will open the next envelope in sequence to determine group allocation and Study ID."

11a) If done, who was blinded after assignment to interventions (for example, participants, care providers, those assessing outcomes) and how

NPT: Whether or not administering co-interventions were blinded to group assignment

11a-i) Specify who was blinded, and who wasn't

Specify who was blinded, and who wasn't. Usually, in web-based trials it is not possible to blind the participants [1, 3] (this should be clearly acknowledged), but it may be possible to blind outcome assessors, those doing data analysis or those administering co-interventions (if any).

	1	2	3	4	5	
subitem not at all important	○	○	○	○	◉	essential

Clear selection

Does your paper address subitem 11a-i? *

Copy and paste relevant sections from the manuscript (include quotes in quotation marks "like this" to indicate direct quotes from your manuscript), or elaborate on this item by providing additional information not in the ms, or briefly explain why the item is not applicable/relevant for your study

The HDRS-24 blinded assessor rater is blinded to participant group assignment. Other research team members are not blinded.

11a-ii) Discuss e.g., whether participants knew which intervention was the “intervention of interest” and which one was the “comparator”

Informed consent procedures (4a-ii) can create biases and certain expectations - discuss e.g., whether participants knew which intervention was the “intervention of interest” and which one was the “comparator”.

	1	2	3	4	5	
subitem not at all important	◉	○	○	○	○	essential

Clear selection

Does your paper address subitem 11a-ii?

Copy and paste relevant sections from the manuscript (include quotes in quotation marks "like this" to indicate direct quotes from your manuscript), or elaborate on this item by providing additional information not in the ms, or briefly explain why the item is not applicable/relevant for your study

Both interventions are of interest.

11b) If relevant, description of the similarity of interventions

(this item is usually not relevant for ehealth trials as it refers to similarity of a placebo or sham intervention to a active medication/intervention)

Does your paper address CONSORT subitem 11b? *

Copy and paste relevant sections from the manuscript (include quotes in quotation marks "like this" to indicate direct quotes from your manuscript), or elaborate on this item by providing additional information not in the ms, or briefly explain why the item is not applicable/relevant for your study

The similarities of interventions is discussed in the paragraphs labeled "Interventions"

12a) Statistical methods used to compare groups for primary and secondary outcomes

NPT: When applicable, details of whether and how the clustering by care providers or centers was addressed

Does your paper address CONSORT subitem 12a? *

Copy and paste relevant sections from the manuscript (include quotes in quotation marks "like this" to indicate direct quotes from your manuscript), or elaborate on this item by providing additional information not in the ms, or briefly explain why the item is not applicable/relevant for your study

"The primary analysis will be a Bayesian analysis of covariance (ANCOVA), with change in BDI-II between baseline and 16 weeks as the outcome and two covariates, the baseline score and the intervention group variable, coded as O (for online), and I (for in-office). We refer to the parameter of interest as Δ, the (baseline-adjusted) difference between the two groups in the change in BDI-II from baseline to 16 weeks, coded such that Δ < 0 means that the on-line group has a smaller decrease in the level of depression over the course of the study. Then, if Δ > -3, the improvement in the on-line group is at most 3 points worse than the improvement in the in-office group and the online group is non-inferior to the in-office group. Using non-informative priors for all study parameters in the ANCOVA model, we will compute and plot the full posterior distribution of Δ, presenting the lower 95% credible interval for Δ, and then calculating the posterior probability that Δ > -3, i.e., the probability (after observing the study data) that on-line CBT is non-inferior, according to our definition. One advantage of a Bayesian approach is that it allows the assigning of posterior probabilities of non-inferiority at other margins, notably other margins near 3 may have equal levels of evidentiary support. For example, without a penalty for multiple testing, we can compute the probability that Δ > -2 or Δ > -4. Another advantage of the Bayesian approach is that it involves a more useful presentation of results than a simple confidence interval or p-value. Finally, the outputs of the Bayesian model can be used as probabilistic inputs for the economic analysis. Since this is a non-inferiority approach, the primary analysis will be per protocol, which in the presence of intervention non-adherence is more conservative than intention-to-treat (i.e., less likely to conclude groups are similar when they are not). Three secondary analyses of the primary outcome will be conducted. First, we will make the above between-group comparison of the changes in BDI-II scores from baseline to each follow-up time point. Secondly, we will perform an intention-to-treat analysis, including outcomes on participants who were non-adherent to their program (standard care CBT vs. CBT online) using established cut-offs. Finally, to assess for sensitivity to random imbalance in baseline characteristic, the following covariates will be added to the ANCOVA model: age, baseline anxiety (BAI), baseline depression (HDRS-24), baseline pain, and baseline mindfulness. If more than 5% of participants included in the per protocol are missing outcome data, that analysis will use multiple imputation within the Bayesian model with full baseline covariate data used to impute missing outcomes. In a second approach, we will assume missing follow-up data are missing not-at-random and replace missing values by values randomly sampled from the upper half (high BDI-II) of their predictive distribution. This represents the assumption that those who have no follow-up BDI-II all have values lower than the median predicted by their baseline characteristics. This will be repeated within the intention-to-treat analysis, where missing outcome data will be more common.

Secondary Outcomes

A similar modeling approach will be used for each secondary outcome (Beck Anxiety Inventory, Quick Inventory of Depressive Symptomatology; (QIDS) and the 24-item Hamilton Depression Rating Scale (HDRS-24), mindfulness (Five-Facet Mindfulness Questionnaire), pain (Brief Pain Inventory), Health Care Cost Diary for Major Depression and, quality of life (EQ-5D). While non-inferiority margins per outcome are non-existent, we will present

(EQ-5D). While non-inferiority margins per outcome are non-existent, we will present probabilities for a range of margins based on fractions of the minimally clinical important difference (MCID) for these scales (where available) or fractions of a standard deviation. All primary and secondary outcomes will be analyzed as continuous variables, with outcomes modelled as normal, t, rescaled beta, log-normal or gamma distributions, whichever is most appropriate."

12a-i) Imputation techniques to deal with attrition / missing values

Imputation techniques to deal with attrition / missing values: Not all participants will use the intervention/comparator as intended and attrition is typically high in ehealth trials. Specify how participants who did not use the application or dropped out from the trial were treated in the statistical analysis (a complete case analysis is strongly discouraged, and simple imputation techniques such as LOCF may also be problematic [4]).

	1	2	3	4	5	
subitem not at all important	○	○	○	○	◉	essential

Clear selection

Does your paper address subitem 12a-i? *

Copy and paste relevant sections from the manuscript (include quotes in quotation marks "like this" to indicate direct quotes from your manuscript), or elaborate on this item by providing additional information not in the ms, or briefly explain why the item is not applicable/relevant for your study

"Missing Data
Missing data may occur when participants miss an assessment or outcome measure but continue study participation or because participants drop out prematurely. In the first case, regression-based imputation at the individual patient level will be used to impute the missing outcome(s). In the second case, imputing data using bottom quintile scores of responders will be used as a worst-case sensitivity analysis46, 47. Loss to follow-up is unavoidable in MDD studies and can reflect poor intervention response. It was a relevant comparison variable in the recently completed RCT where loss to follow-up in the intervention arm was 10%, compared to 60% in the standard psychiatry control arm17,18. While differences may be more modest in the proposed trial (e.g. 40% in group in-office CBT versus 10% in group online CBT) they will be carefully monitored and, as previously mentioned, represented in the cost-effectiveness and intention-to-treat analyses."

12b) Methods for additional analyses, such as subgroup analyses and adjusted analyses

Does your paper address CONSORT subitem 12b? *

Copy and paste relevant sections from the manuscript (include quotes in quotation marks "like this" to indicate direct quotes from your manuscript), or elaborate on this item by providing additional information not in the ms, or briefly explain why the item is not applicable/relevant for your study

"Cost-effectiveness
We will conduct a full economic evaluation following the design of a cost-utility analysis conducted from a societal and healthcare consumer/payer's perspective. We will adopt the following time horizons: within-trial (4 and 10 months) and the lifetime of the trial cohort. A within-trial cost utility analysis will focus only on interventions directly evaluated in the trial54. We will estimate costs for: i) the interventions; ii) physician services; iii) emergency department visits and hospitalizations; iv) outpatient diagnostic tests; v) drugs, including drug unrelated to major depressive disorder; vi) home care; vii) long-term care; viii) out-of-pocket costs; and ix) productivity costs. The intervention will be cost-estimated by estimating the value of time of those administering the intervention, facility-use costs, and device/equipment costs, amortized over an appropriate period. Resource utilization, out-of-pocket costs, and productivity costs will be estimated using a patient cost diary. We will assess health outcome data in QALYs using the EQ-5D at each time point48-51. Cumulative costs and QALYs will be estimated and compared in order to calculate the incremental cost utility ratio, and incremental net health benefit. We will plot the cost-effectiveness acceptability curves and confidence ellipses to demonstrate variability in a trial sample and illustrate probability of online CBT-M being cost-effective compared with standard CBT program at a range of willingness to pay thresholds."

X26) REB/IRB Approval and Ethical Considerations [recommended as subheading under "Methods"] (not a CONSORT item)

X26-i) Comment on ethics committee approval

	1	2	3	4	5	
subitem not at all important	(●)	( )	( )	( )	( )	essential

Clear selection

Does your paper address subitem X26-i?

Copy and paste relevant sections from the manuscript (include quotes in quotation marks "like this" to indicate direct quotes from your manuscript), or elaborate on this item by providing additional information not in the ms, or briefly explain why the item is not applicable/relevant for your study

Your answer

x26-ii) Outline informed consent procedures

Outline informed consent procedures e.g., if consent was obtained offline or online (how? Checkbox, etc.?), and what information was provided (see 4a-ii). See [6] for some items to be included in informed consent documents.

	1	2	3	4	5	
subitem not at all important	(•)	( )	( )	( )	( )	essential

Clear selection

Does your paper address subitem X26-ii?

Copy and paste relevant sections from the manuscript (include quotes in quotation marks "like this" to indicate direct quotes from your manuscript), or elaborate on this item by providing additional information not in the ms, or briefly explain why the item is not applicable/relevant for your study

Your answer

X26-iii) Safety and security procedures

Safety and security procedures, incl. privacy considerations, and any steps taken to reduce the likelihood or detection of harm (e.g., education and training, availability of a hotline)

	1	2	3	4	5	
subitem not at all important	(•)	( )	( )	( )	( )	essential

Clear selection

Does your paper address subitem X26-iii?

Copy and paste relevant sections from the manuscript (include quotes in quotation marks "like this" to indicate direct quotes from your manuscript), or elaborate on this item by providing additional information not in the ms, or briefly explain why the item is not applicable/relevant for your study

Your answer

RESULTS

13a) For each group, the numbers of participants who were randomly assigned, received intended treatment, and were analysed for the primary outcome

NPT: The number of care providers or centers performing the intervention in each group and the number of patients treated by each care provider in each center

Does your paper address CONSORT subitem 13a? *

Copy and paste relevant sections from the manuscript (include quotes in quotation marks "like this" to indicate direct quotes from your manuscript), or elaborate on this item by providing additional information not in the ms, or briefly explain why the item is not applicable/relevant for your study

N/A

Your answer must have a minimum of 25 characters.

13b) For each group, losses and exclusions after randomisation, together with reasons

Does your paper address CONSORT subitem 13b? (NOTE: Preferably, this is shown in a CONSORT flow diagram) *

Copy and paste relevant sections from the manuscript (include quotes in quotation marks "like this" to indicate direct quotes from your manuscript), or elaborate on this item by providing additional information not in the ms, or briefly explain why the item is not applicable/relevant for your study

Not applicable - results have not been obtained as this is a protocol paper.

13b-i) Attrition diagram

Strongly recommended: An attrition diagram (e.g., proportion of participants still logging in or using the intervention/comparator in each group plotted over time, similar to a survival curve) or other figures or tables demonstrating usage/dose/engagement.

	1	2	3	4	5	
subitem not at all important	(•)	( )	( )	( )	( )	essential

Clear selection

Does your paper address subitem 13b-i?

Copy and paste relevant sections from the manuscript or cite the figure number if applicable (include quotes in quotation marks "like this" to indicate direct quotes from your manuscript), or elaborate on this item by providing additional information not in the ms, or briefly explain why the item is not applicable/relevant for your study

Your answer

14a) Dates defining the periods of recruitment and follow-up

Does your paper address CONSORT subitem 14a? *

Copy and paste relevant sections from the manuscript (include quotes in quotation marks "like this" to indicate direct quotes from your manuscript), or elaborate on this item by providing additional information not in the ms, or briefly explain why the item is not applicable/relevant for your study

"The present study has 3 aims: (1) to evaluate whether online group CBT-M plus standard psychiatric care is non-inferior in efficacy to office-based group CBT-M plus standard care as measured by Beck Depression Inventory-II (BDI-II) score in the treatment of adults with major depressive disorder (MDD); (2) to evaluate whether online group CBT-M is more cost-effective than office-based group CBT-M at post-intervention and 6 months post-intervention follow-up; (3) to assess, within the online group CBT-M intervention group, whether digitally recorded adherence data (i.e. online workbooks completed, Fitbit tracked steps, online text messages exchanged, phone sessions completed) predict outcome benefits as indicated by changed BDI-II score."

14a-i) Indicate if critical “secular events” fell into the study period

Indicate if critical “secular events” fell into the study period, e.g., significant changes in Internet resources available or “changes in computer hardware or Internet delivery resources”

	1	2	3	4	5	
subitem not at all important	(●)	( )	( )	( )	( )	essential

Clear selection

Does your paper address subitem 14a-i?

Copy and paste relevant sections from the manuscript (include quotes in quotation marks "like this" to indicate direct quotes from your manuscript), or elaborate on this item by providing additional information not in the ms, or briefly explain why the item is not applicable/relevant for your study

Your answer

14b) Why the trial ended or was stopped (early)

Does your paper address CONSORT subitem 14b? *

Copy and paste relevant sections from the manuscript (include quotes in quotation marks "like this" to indicate direct quotes from your manuscript), or elaborate on this item by providing additional information not in the ms, or briefly explain why the item is not applicable/relevant for your study

This is not applicable as this is a protocol paper and this information is not yet available.

15) A table showing baseline demographic and clinical characteristics for each group

NPT: When applicable, a description of care providers (case volume, qualification, expertise, etc.) and centers (volume) in each group

Does your paper address CONSORT subitem 15? *

Copy and paste relevant sections from the manuscript (include quotes in quotation marks "like this" to indicate direct quotes from your manuscript), or elaborate on this item by providing additional information not in the ms, or briefly explain why the item is not applicable/relevant for your study

This is not applicable as this is a protocol paper and this information is not yet available.

15-i) Report demographics associated with digital divide issues

In ehealth trials it is particularly important to report demographics associated with digital divide issues, such as age, education, gender, social-economic status, computer/Internet/ehealth literacy of the participants, if known.

	1	2	3	4	5	
subitem not at all important	(●)	○	○	○	○	essential

Clear selection

Does your paper address subitem 15-i? *

Copy and paste relevant sections from the manuscript (include quotes in quotation marks "like this" to indicate direct quotes from your manuscript), or elaborate on this item by providing additional information not in the ms, or briefly explain why the item is not applicable/relevant for your study

This is not applicable as this is a protocol paper and this information is not yet available.

16) For each group, number of participants (denominator) included in each analysis and whether the analysis was by original assigned groups

16-i) Report multiple “denominators” and provide definitions

Report multiple “denominators” and provide definitions: Report N’s (and effect sizes) “across a range of study participation [and use] thresholds” [1], e.g., N exposed, N consented, N used more than x times, N used more than y weeks, N participants “used” the intervention/comparator at specific pre-defined time points of interest (in absolute and relative numbers per group). Always clearly define “use” of the intervention.

	1	2	3	4	5	
subitem not at all important	(●)	○	○	○	○	essential

Clear selection

Does your paper address subitem 16-i? *

Copy and paste relevant sections from the manuscript (include quotes in quotation marks "like this" to indicate direct quotes from your manuscript), or elaborate on this item by providing additional information not in the ms, or briefly explain why the item is not applicable/relevant for your study

This is not applicable as this is a protocol paper and this information is not yet available.

16-ii) Primary analysis should be intent-to-treat

Primary analysis should be intent-to-treat, secondary analyses could include comparing only “users”, with the appropriate caveats that this is no longer a randomized sample (see 18-i).

	1	2	3	4	5	
subitem not at all important	○	○	○	○	◉	essential

Clear selection

Does your paper address subitem 16-ii?

Copy and paste relevant sections from the manuscript (include quotes in quotation marks "like this" to indicate direct quotes from your manuscript), or elaborate on this item by providing additional information not in the ms, or briefly explain why the item is not applicable/relevant for your study

Your answer

17a) For each primary and secondary outcome, results for each group, and the estimated effect size and its precision (such as 95% confidence interval)

Does your paper address CONSORT subitem 17a? *

Copy and paste relevant sections from the manuscript (include quotes in quotation marks "like this" to indicate direct quotes from your manuscript), or elaborate on this item by providing additional information not in the ms, or briefly explain why the item is not applicable/relevant for your study

This is not applicable as this is a protocol paper and this information is not yet available.

17a-i) Presentation of process outcomes such as metrics of use and intensity of use

In addition to primary/secondary (clinical) outcomes, the presentation of process outcomes such as metrics of use and intensity of use (dose, exposure) and their operational definitions is critical. This does not only refer to metrics of attrition (13-b) (often a binary variable), but also to more continuous exposure metrics such as "average session length". These must be accompanied by a technical description how a metric like a "session" is defined (e.g., timeout after idle time) [1] (report under item 6a).

	1	2	3	4	5	
subitem not at all important	(●)	( )	( )	( )	( )	essential

Clear selection

Does your paper address subitem 17a-i?

Copy and paste relevant sections from the manuscript (include quotes in quotation marks "like this" to indicate direct quotes from your manuscript), or elaborate on this item by providing additional information not in the ms, or briefly explain why the item is not applicable/relevant for your study

Your answer

17b) For binary outcomes, presentation of both absolute and relative effect sizes is recommended

Does your paper address CONSORT subitem 17b? *

Copy and paste relevant sections from the manuscript (include quotes in quotation marks "like this" to indicate direct quotes from your manuscript), or elaborate on this item by providing additional information not in the ms, or briefly explain why the item is not applicable/relevant for your study

This is not applicable as this is a protocol paper and this information is not yet available.

18) Results of any other analyses performed, including subgroup analyses and adjusted analyses, distinguishing pre-specified from exploratory

Does your paper address CONSORT subitem 18? *

Copy and paste relevant sections from the manuscript (include quotes in quotation marks "like this" to indicate direct quotes from your manuscript), or elaborate on this item by providing additional information not in the ms, or briefly explain why the item is not applicable/relevant for your study

This is not applicable as this is a protocol paper and this information is not yet available.

18-i) Subgroup analysis of comparing only users

A subgroup analysis of comparing only users is not uncommon in ehealth trials, but if done, it must be stressed that this is a self-selected sample and no longer an unbiased sample from a randomized trial (see 16-iii).

	1	2	3	4	5	
subitem not at all important	(•)	( )	( )	( )	( )	essential

Clear selection

Does your paper address subitem 18-i?

Copy and paste relevant sections from the manuscript (include quotes in quotation marks "like this" to indicate direct quotes from your manuscript), or elaborate on this item by providing additional information not in the ms, or briefly explain why the item is not applicable/relevant for your study

Your answer

19) All important harms or unintended effects in each group

(for specific guidance see CONSORT for harms)

Does your paper address CONSORT subitem 19? *

Copy and paste relevant sections from the manuscript (include quotes in quotation marks "like this" to indicate direct quotes from your manuscript), or elaborate on this item by providing additional information not in the ms, or briefly explain why the item is not applicable/relevant for your study

This is addressed in the consent discussion where informed consent is obtained prior to study participation. Please see above.

19-i) Include privacy breaches, technical problems

Include privacy breaches, technical problems. This does not only include physical "harm" to participants, but also incidents such as perceived or real privacy breaches [1], technical problems, and other unexpected/unintended incidents. "Unintended effects" also includes unintended positive effects [2].

	1	2	3	4	5	
subitem not at all important	(●)	○	○	○	○	essential

Clear selection

Does your paper address subitem 19-i?

Copy and paste relevant sections from the manuscript (include quotes in quotation marks "like this" to indicate direct quotes from your manuscript), or elaborate on this item by providing additional information not in the ms, or briefly explain why the item is not applicable/relevant for your study

Your answer

19-ii) Include qualitative feedback from participants or observations from staff/researchers

Include qualitative feedback from participants or observations from staff/researchers, if available, on strengths and shortcomings of the application, especially if they point to unintended/unexpected effects or uses. This includes (if available) reasons for why people did or did not use the application as intended by the developers.

	1	2	3	4	5	
subitem not at all important	(●)	( )	( )	( )	( )	essential

Clear selection

Does your paper address subitem 19-ii?

Copy and paste relevant sections from the manuscript (include quotes in quotation marks "like this" to indicate direct quotes from your manuscript), or elaborate on this item by providing additional information not in the ms, or briefly explain why the item is not applicable/relevant for your study

Your answer

DISCUSSION

22) Interpretation consistent with results, balancing benefits and harms, and considering other relevant evidence

NPT: In addition, take into account the choice of the comparator, lack of or partial blinding, and unequal expertise of care providers or centers in each group

22-i) Restate study questions and summarize the answers suggested by the data, starting with primary outcomes and process outcomes (use)

Restate study questions and summarize the answers suggested by the data, starting with primary outcomes and process outcomes (use).

	1	2	3	4	5	
subitem not at all important	◉	○	○	○	○	essential

Clear selection

Does your paper address subitem 22-i? *

Copy and paste relevant sections from the manuscript (include quotes in quotation marks "like this" to indicate direct quotes from your manuscript), or elaborate on this item by providing additional information not in the ms, or briefly explain why the item is not applicable/relevant for your study

This is not applicable as this is a protocol paper and this information is not yet available.

22-ii) Highlight unanswered new questions, suggest future research

Highlight unanswered new questions, suggest future research.

	1	2	3	4	5	
subitem not at all important	◉	○	○	○	○	essential

Clear selection

Does your paper address subitem 22-ii?

Copy and paste relevant sections from the manuscript (include quotes in quotation marks "like this" to indicate direct quotes from your manuscript), or elaborate on this item by providing additional information not in the ms, or briefly explain why the item is not applicable/relevant for your study

This is not applicable as this is a protocol paper and this information is not yet available.

20) Trial limitations, addressing sources of potential bias, imprecision, and, if relevant, multiplicity of analyses

20-i) Typical limitations in ehealth trials

Typical limitations in ehealth trials: Participants in ehealth trials are rarely blinded. Ehealth trials often look at a multiplicity of outcomes, increasing risk for a Type I error. Discuss biases due to non-use of the intervention/usability issues, biases through informed consent procedures, unexpected events.

	1	2	3	4	5	
subitem not at all important	(●)	( )	( )	( )	( )	essential

Clear selection

Does your paper address subitem 20-i? *

Copy and paste relevant sections from the manuscript (include quotes in quotation marks "like this" to indicate direct quotes from your manuscript), or elaborate on this item by providing additional information not in the ms, or briefly explain why the item is not applicable/relevant for your study

This is not applicable as this is a protocol paper and this information is not yet available.

21) Generalisability (external validity, applicability) of the trial findings

NPT: External validity of the trial findings according to the intervention, comparators, patients, and care providers or centers involved in the trial

21-i) Generalizability to other populations

Generalizability to other populations: In particular, discuss generalizability to a general Internet population, outside of a RCT setting, and general patient population, including applicability of the study results for other organizations

	1	2	3	4	5	
subitem not at all important	(•)	( )	( )	( )	( )	essential

Clear selection

Does your paper address subitem 21-i?

Copy and paste relevant sections from the manuscript (include quotes in quotation marks "like this" to indicate direct quotes from your manuscript), or elaborate on this item by providing additional information not in the ms, or briefly explain why the item is not applicable/relevant for your study

This is not applicable as this is a protocol paper and this information is not yet available.

21-ii) Discuss if there were elements in the RCT that would be different in a routine application setting

Discuss if there were elements in the RCT that would be different in a routine application setting (e.g., prompts/reminders, more human involvement, training sessions or other co-interventions) and what impact the omission of these elements could have on use, adoption, or outcomes if the intervention is applied outside of a RCT setting.

	1	2	3	4	5	
subitem not at all important	(•)	( )	( )	( )	( )	essential

Clear selection

Does your paper address subitem 21-ii?

Copy and paste relevant sections from the manuscript (include quotes in quotation marks "like this" to indicate direct quotes from your manuscript), or elaborate on this item by providing additional information not in the ms, or briefly explain why the item is not applicable/relevant for your study

This is not applicable as this is a protocol paper and this information is not yet available.

OTHER INFORMATION

23) Registration number and name of trial registry

Does your paper address CONSORT subitem 23? *

Copy and paste relevant sections from the manuscript (include quotes in quotation marks "like this" to indicate direct quotes from your manuscript), or elaborate on this item by providing additional information not in the ms, or briefly explain why the item is not applicable/relevant for your study

ClinicalTrials.gov Identifier: NCT04825535

24) Where the full trial protocol can be accessed, if available

Does your paper address CONSORT subitem 24? *

Cite a Multimedia Appendix, other reference, or copy and paste relevant sections from the manuscript (include quotes in quotation marks "like this" to indicate direct quotes from your manuscript), or elaborate on this item by providing additional information not in the ms, or briefly explain why the item is not applicable/relevant for your study

Not available for public access

25) Sources of funding and other support (such as supply of drugs), role of funders

Does your paper address CONSORT subitem 25? *

Copy and paste relevant sections from the manuscript (include quotes in quotation marks "like this" to indicate direct quotes from your manuscript), or elaborate on this item by providing additional information not in the ms, or briefly explain why the item is not applicable/relevant for your study

The study is funded by the Canadian Institute of Health Research (CIHR).

X27) Conflicts of Interest (not a CONSORT item)

X27-i) State the relation of the study team towards the system being evaluated

In addition to the usual declaration of interests (financial or otherwise), also state the relation of the study team towards the system being evaluated, i.e., state if the authors/evaluators are distinct from or identical with the developers/sponsors of the intervention.

	1	2	3	4	5	
subitem not at all important	◉	○	○	○	○	essential

Clear selection

Does your paper address subitem X27-i?

Copy and paste relevant sections from the manuscript (include quotes in quotation marks "like this" to indicate direct quotes from your manuscript), or elaborate on this item by providing additional information not in the ms, or briefly explain why the item is not applicable/relevant for your study

Your answer

About the CONSORT EHEALTH checklist

As a result of using this checklist, did you make changes in your manuscript? *

- ○ yes, major changes
- ○ yes, minor changes
- ◉ no

What were the most important changes you made as a result of using this checklist?

Your answer

How much time did you spend on going through the checklist INCLUDING making changes in your manuscript *

Around five hours were spent.

As a result of using this checklist, do you think your manuscript has improved? *

- ◉ yes
- ○ no
- ○ Other:

Would you like to become involved in the CONSORT EHEALTH group?

This would involve for example becoming involved in participating in a workshop and writing an "Explanation and Elaboration" document

- ○ yes
- ◉ no
- ○ Other:

Clear selection

Any other comments or questions on CONSORT EHEALTH

Your answer

STOP - Save this form as PDF before you click submit

To generate a record that you filled in this form, we recommend to generate a PDF of this page (on a Mac, simply select "print" and then select "print as PDF") before you submit it.

When you submit your (revised) paper to JMIR, please upload the PDF as supplementary file.

Don't worry if some text in the textboxes is cut off, as we still have the complete information in our database. Thank you!

Final step: Click submit !

Click submit so we have your answers in our database!

Submit Clear form

Never submit passwords through Google Forms.

This content is neither created nor endorsed by Google. Report Abuse - Terms of Service - Privacy Policy

Google Forms
